# Supplementary material for: Consumer-driven nutrient recycling of freshwater decapods: Linking ecological theories and application in integrated multitrophic aquaculture
Source: PLoS One. 2023 Oct 26;18(10):e0262972. doi: 10.1371/journal.pone.0262972 (PMC10602317; doi:10.1371/journal.pone.0262972)
Supplement: S1 Table — (DOCX) [file pone.0262972.s001.docx]

|  | AIC |
| --- | --- |
| ***M. borellii*** |  |
| - Excretion N |  |
| ~ Feed*Body mass + Feed*N body + N body*Body mass | -35.68 |
| ~ Feed + Body mass + N body + Feed*Body mass + N body*Body mass | -37.55 |
| ~ Feed + Body mass + N body + N body*Body mass | -38.23 |
| ~ Body mass + N body + N body*Body mass | -39.61 |
| ~ Body mass + N body | -40.55 |
|  |  |
| - Excretion P |  |
| ~ Feed*Body mass + Feed*P body + P body*Body mass | -44.59 |
| ~ Feed + Body mass + P body + Feed*Body mass + Feed*P body | -46.50 |
| ~ Feed + Body mass + P body + Feed*P body | -48.26 |
|  |  |
| - Excretion N:P |  |
| ~ Feed*Body mass + Feed*N:P body + N:P body*Body mass | -29.24 |
|  |  |
| ***A. uruguayana*** |  |
| - Excretion N |  |
| **~** Feed*Body mass + Feed*N body + N body*Body mass | -36.81 |
| ~ Feed + Body mass + N body + Feed*Body mass + Feed*N body | -38.23 |
|  |  |
| - Excretion P |  |
| ~ Feed*Body mass + Feed*P body + P body*Body mass | -35.80 |
| ~ Feed + Body mass + P body + Feed*Body mass + P body*Body mass | -37.69 |
|  |  |
| - Excretion N:P |  |
| ~ Feed*Body mass + Feed*N:P body + N:P body*Body mass | -22.94 |
| ~ Feed + Body mass + N:P body + Feed*N:P body + N:P body*Body mass | -24.90 |
|  |  |
| ***T. borellianus*** |  |
| - Excretion N |  |
| ~ Feed*Body mass + Feed*N body + N body*Body mass | -37.47 |
| ~ Feed + Body mass + N body + Feed*Body mass + N body*Body mass | -39.45 |
|  |  |
| - Excretion P |  |
| ~ Feed*Body mass + Feed*P body + P body*Body mass | 2.02 |
| ~ Feed + Body mass + P body + Feed*Body mass + P body*Body mass | 0.36 |
|  |  |
| - Excretion N:P | - |
|  |  |
