# Supplementary material for: Consumer-driven nutrient recycling of freshwater decapods: Linking ecological theories and application in integrated multitrophic aquaculture
Source: PLoS One. 2023 Oct 26;18(10):e0262972. doi: 10.1371/journal.pone.0262972 (PMC10602317; doi:10.1371/journal.pone.0262972)
Supplement: Attachment — Submitted filename: Plos Review.pdf [file pone.0262972.s003.pdf]

This is a review of the revised manuscript, “Consumer-driven nutrient recycling of freshwater decapods: linking ecological theories and application in integrated multi-trophic aquaculture”

The Introduction of this paper is a fine review of CND and I suggest clearly linking this review to CND in an aquaculture setting. There is too much detail (even in the revised version) in the literature review that is not related to CND in aquaculture contexts. My suggestion is to condense the first three or four paragraphs into the key points of CND (i.e., EST, MTE) and explicitly state that CND has been used almost exclusively in natural (more or less) ecological settings testing the role of animals in ecosystems, but its use in applied setting such as aquaculture is limited. I think it is possible to do this in one paragraph with reference to Atkinson et al 2017, Vanni and McIntyre 2016, Brown et al 2008, and Sterner and Else 2002) for each point, respectively. Then move into the more specific description of the region and goals of the study.

The species descriptions were difficult to follow. I suggest a conceptual figure highlighting hypothesized relationships among food, body, excretion N:P based on this description for each species. This would be a helpful reference to readers. This would also help to explain better the predictions related to how each species will respond to food treatments.

The table with food content information is useful. I suggest explaining how the proximal contents are related to C,N, and P in the introduction/conceptual model. This link is not made in the current Introduction.

I think the data are over analyzed. The correlation and PCA procedures appear to produce similar patterns. The PCA seems better suited for the supplement but should be referenced first. The same data are presented several ways. I suggest finding the simplest and most intuitive way to present the key findings. With so many figures I was confused about what the authors thought were the key findings. Additionally, I suggest conducting a multivariate analysis of co-variance (MANCOVA) rather than several separate ANOVAs, regressions, and ANCOVAs. I suggest following Benstead, J.P., W.F. Cross, J.G. March, W.H. McDowell, A. Ramírez, and A.P. Covich. 2010. Biotic and abiotic controls on the ecosystem significance of consumer excretion in two contrasting tropical streams. Freshwater Biology 55:2047-2061. PDF is attached to my comments in the journal portal system

Line 20: Should productive be changed to aquaculture production systems? This is a comment that applies throughout

Line 43: I suggest rephrasing this. The citation formatting of Plos is not well suited for this kind of reference. The meaning is lost here.

Line 73: I’d suggest deleting this. It is important to consider. However, it is unclear how this is related to the goals of the paper. If the authors wish to bring this up, I’d move it to the discussion and link to the result of the paper.

Line 88 I suggest combining paragraph starting on line 88 and line 100.

Line 108. I recommend providing a simple example of this. It is not clear how crustaceans would be used in this setting. “The use of crustaceans to……in aquaculture settings is growing.”. For most readers, aquaculture is seen as producing these animals and I do not think that is what is happening here.

Line 108 and 109 are redundant.

Line 118: I suggest removing this sentence. It is not needed.

Line 126: These are clearly explained. The manner in which these are explained interrupts the flow of the paper. I invite the authros to re write this section such that questions are followed by “we hypothesized….. because…..” this would be easier for the reader to follow along with the logical

With this in mind, the authors should consider explicitly stating what feed manipulations they are considering and ensure that enough background information is provided in the literature review. As is, there is very vague information on CND, but they are manipulating protein content etc which are much more specific. This should be made clear to the reader early to help the reader understand the logic of the predictions the authors make.

Line 174: I am not sure this is the correct use of “elaborated”. I suggest using a different word.

Line 176: Do the authors mean the discharged water was subsampled? There are a few words in this section that are not quite correct.

Report fewer significant digits for F and P values in the results. I’d also suggest reporting degrees of freedom for each test. PCA should be a supplemental figure.

Line 412: I realize the authors mention expressing mass specific excretion rates as “ Excretion throughout the paper” or something to that effect. My suggestion is to stick with mass-specific excretion because the negative relationship is expected to occur. It is slightly confusing to read this in the abstract and discussion because the opposite pattern is true for per capita rates. My preference is to be clear throughout the paper by using mass-specific rates..

Line 419: I suggest explaining this briefly. “These results did not support the GRH because…..

I’d also suggest ending this with a stronger conclusion about the work.

Line 420 -429 This is repeating results. Id suggest placing the results into the broader context of ES and MTE. Is this result expected? How does this compared to what others have found in decapods elsewhere? Why does this matter to Aquaculture settings?

Line 432 Consider removing this sentence. It is not needed.

Line 452: This paragraph is narrowly focused. Consider broadening this from decapods to other inverts/vertebrates. Is this within the rage of expected values? Does this further support for MTE? This last sentence is a good topic for discussion and needs to be expanded upon <u>why</u> this is explained by MTE.

Line 461 This paragraph does a good job of placing results in the context of the literature

The last paragraph is very good. I think that integrating this information throughout the previous discussion sections would improve the discussion. I think this is because the current version of the MS is too focused on explain EST and MTE. This is already well established. The value of this paper is putting those theories into application and practice. This last section is really the principle goal of the paper. There are 11 short sections prior this that essentially repeat the results. My suggestion is to consider the key results in the context of these last four paragraphs. There are 16 paragraphs (sections) in the discussion. That is too many. This can be solved by focusing the results into this interesting last section. To me this applied EST is the most entire goal of the paper. That gets lost in the previous sections.
