# Supplementary material for: Consumer-driven nutrient recycling of freshwater decapods: Linking ecological theories and application in integrated multitrophic aquaculture
Source: PLoS One. 2023 Oct 26;18(10):e0262972. doi: 10.1371/journal.pone.0262972 (PMC10602317; doi:10.1371/journal.pone.0262972)
Supplement: Attachment — Submitted filename: Benstead et al 2010.pdf [file pone.0262972.s004.pdf]

Freshwater Biology

Freshwater Biology (2010) **55**, 2047–2061 doi:10.1111/j.1365-2427.2010.02461.x

Biotic and abiotic controls on the ecosystem significance of consumer excretion in two contrasting tropical streams

JONATHAN P. BENSTEAD*, WYATT F. CROSS†, JAMES G. MARCH‡, WILLIAM H. McDOWELL§, ALONSO RAMÍREZ¶ AND ALAN P. COVICH†

*Department of Biological Sciences, University of Alabama, Tuscaloosa, AL, U.S.A.
†Odum School of Ecology, University of Georgia, Athens, Georgia, GA, U.S.A.
‡Biology Department, Washington and Jefferson College, Washington, PA, U.S.A.
§Department of Natural Resources and the Environment, University of New Hampshire, Durham, NH, U.S.A.
¶Institute for Tropical Ecosystem Studies, University of Puerto Rico, San Juan, PR, Puerto Rico

SUMMARY

1. Excretion of nitrogen (N) and phosphorus (P) is a direct and potentially important role for aquatic consumers in nutrient cycling that has recently garnered increased attention. The ecosystem-level significance of excreted nutrients depends on a suite of abiotic and biotic factors, however, and few studies have coupled measurements of excretion with consideration of its likely importance for whole-system nutrient fluxes.
2. We measured rates and ratios of N and P excretion by shrimps (*Xiphocaris elongata* and *Atya* spp.) in two tropical streams that differed strongly in shrimp biomass because a waterfall excluded predatory fish from one site. We also made measurements of shrimp and basal resource carbon (C), N and P content and estimated shrimp densities and ecosystem-level N and P excretion and uptake. Finally, we used a 3-year record of discharge and NH_4-N concentration in the high-biomass stream to estimate temporal variation in the distance required for excretion to turn over the ambient NH_4-N pool.
3. Per cent C, N, and P body content of *Xiphocaris* was significantly higher than that of *Atya*. Only per cent P body content showed significant negative relationships with body mass. C:N of *Atya* increased significantly with body mass and was higher than that of *Xiphocaris*. N : P of *Xiphocaris* was significantly higher than that of *Atya*.
4. Excretion rates ranged from 0.16–3.80 μmol NH_4-N shrimp^{-1} h^{-1}, 0.23–5.76 μmol total dissolved nitrogen (TDN) shrimp^{-1} h^{-1} and 0.002–0.186 μmol total dissolved phosphorus (TDP) shrimp^{-1} h^{-1}. Body size was generally a strong predictor of excretion rates in both taxa, differing between *Xiphocaris* and *Atya* for TDP but not NH_4-N and TDN. Excretion rates showed statistically significant but weak relationships with body content stoichiometry.
5. Large between-stream differences in shrimp biomass drove differences in total excretion by the two shrimp communities (22.3 versus 0.20 μmol NH_4-N m^{-2} h^{-1}, 37.5 versus 0.26 μmol TDN m^{-2} h^{-1} and 1.1 versus 0.015 μmol TDP m^{-2} h^{-1}), equivalent to 21% and 0.5% of NH_4-N uptake and 5% and <0.1% of P uptake measured in the high- and low-biomass stream, respectively. Distances required for excretion to turn over the ambient NH_4-N pool varied more than a hundredfold over the 3-year record in the high-shrimp stream, driven by variability in discharge and NH_4-N concentration.

Correspondence: Jonathan P. Benstead, Department of Biological Sciences, University of Alabama, Tuscaloosa, AL 35487, U.S.A.
E-mail: jbenstead@bama.ua.edu
Present address: Wyatt F. Cross, Department of Ecology, Montana State University, Bozeman, MT 59717, U.S.A.

© 2010 Blackwell Publishing Ltd

6. Our results underscore the importance of both biotic and abiotic factors in controlling consumer excretion and its significance for nutrient cycling in aquatic ecosystems. Differences in community-level excretion rates were related to spatial patterns in shrimp biomass dictated by geomorphology and the presence of predators. Abiotic factors also had important effects through temporal patterns in discharge and nutrient concentrations. Future excretion studies that focus on nutrient cycling should consider both biotic and abiotic factors in assessing the significance of consumer excretion in aquatic ecosystems.

Keywords: consumer-driven nutrient recycling, ecological stoichiometry, El Yunque, Luquillo Experimental Forest, Puerto Rico

Introduction

Animals have direct effects on nutrient cycling in aquatic ecosystems through their excretion of waste products (Kitchell *et al.*, 1979; Vanni, 2002). Inputs of biologically important elements (i.e. nitrogen [N] and phosphorus [P]) from excretion complement and interact with the indirect effects on nutrient cycling mediated by consumers through their consumption and removal of biomass (Knoll *et al.*, 2009). Fluxes of N and P through consumer biomass have long been of interest to aquatic ecologists, from the perspectives of both physiology and nutrient cycling (e.g. Rigler, 1961; Pomeroy, Matthews & Min, 1963). Interest in consumer excretion has recently increased, however, spurred by renewed emphases on ecological stoichiometry and body size (Sterner & Elser, 2002; Woodward *et al.*, 2005; Hall *et al.*, 2007) and the role of excretion in mediating trophic cascades (e.g. Findlay *et al.*, 2005). Despite this ongoing interest, the general significance of consumer excretion for whole-system nutrient cycling in aquatic ecosystems is still unclear (Vanni, 2002; Cross *et al.*, 2005).

The relative magnitude of consumer excretion and its potential significance to ecosystem-level nutrient cycling depend on a number of biotic and abiotic factors. Characteristics of the consumer community are clearly important. Both biomass of consumers and the size structure of their populations control the rate of total nutrient excretion, with small consumers having higher mass-specific rates of excretion than larger individuals (Hall, Tank & Dybdahl, 2003; Hall *et al.*, 2007). Elemental demand (driven by body stoichiometry and constrained by phylogeny) combines with diet nutrient content to control the nutrient ratios of excretion (Vanni *et al.*, 2002; Torres & Vanni, 2007). Although stoichiometric interactions at the level of the individual consumer constrain rates and ratios of excretion, the abiotic environment ultimately sets the template for assessing the relative significance of recycled nutrients. Both the volume and turnover rate of water (relative to consumer biomass) are important, as are ambient nutrient concentrations (McIntyre *et al.*, 2008). Finally, the identity of the limiting nutrient may control the potential significance of consumer excretion for ecosystem processes (Vanni *et al.*, 2002). Biotic and abiotic factors thus interact to control the potential role of consumer excretion as a significant flux in aquatic nutrient cycles (Hall *et al.*, 2007; McIntyre *et al.*, 2008).

Given the potential interactions between these biotic and abiotic factors, populations of shrimps in tropical streams represent useful model systems in which to examine the relative importance of consumer excretion for ecosystem-level nutrient cycling. Shrimps are relatively common components of tropical stream communities, often occurring at high densities and biomass (Covich, 1988b). Their spatial distribution is often patchy at the catchment scale, because of interactions between geomorphology and the relative abundance of their predators. For example, in Puerto Rico, densities of shrimps are highest in those reaches above waterfalls that cannot be accessed by their predators (mostly fish species; Pringle *et al.*, 1999; Covich *et al.*, 2009). In addition to spatial variability in shrimp density, the streams inhabited by these consumer taxa are relatively dynamic systems, in which the abiotic conditions that affect the potential significance of consumer excretion (i.e. discharge and ambient nutrient availability) are extremely variable temporally. In sum, the spatial and temporal variability typical of shrimps and their stream habitat results in the potential for great

variation in the significance of shrimp excretion for nutrient cycling in these systems.

In this study, we measured resource and body stoichiometry (%C, %N and %P) and excretion rates of N and P in two cosmopolitan genera of shrimps (*Atya* and *Xiphocaris*) collected from two tropical Puerto Rican streams that differed greatly in shrimp biomass because of geomorphic exclusion of predators. To assess the significance of shrimp excretion for whole-reach nutrient dynamics, we combined excretion data with estimates of densities for these freshwater shrimp genera (Cross *et al.*, 2008) and compared rates of potential N and P supply by shrimp to the magnitude of ecosystem demand. Finally, we estimated distances required for shrimp excretion to turn over the ambient NH_4-N pool over a 3-year period. We discuss our results with respect to the likely significance of consumer excretion for fluxes and cycling of nitrogen and phosphorus in these tropical streams, as well as in aquatic ecosystems in general.

Methods

Study sites

Fieldwork for this study was conducted in single reaches of two second-order rainforest streams, Quebrada Prieta and Quebrada Bisley-3 (hereafter, Prieta and Bisley, respectively), which drain the Luquillo Long-Term Ecological Research (LTER) site within the Luquillo Experimental Forest (LEF) in northeastern Puerto Rico (also known as the El Yunque National Forest and formerly the Caribbean National Forest). The LEF is characterised as tropical-wet forest and receives an average of 3.5 m of precipitation per year at the altitudes at which these streams are located (Heartsill-Scalley *et al.*, 2007). Long-term averages indicate that May through December is typically wetter than the rest of the year, but this seasonal pattern is highly variable. The two stream reaches are similar in size (mean width 2–3 m) and mean discharge and nutrient chemistry (Table 1). Both streams drain small, steep catchments and are characterised by series of pools interspersed with boulder-lined riffles. Large boulders and cobble dominate the substrata; fine sediments and silt are present in depositional areas of pools and between large boulders. The streams are heavily shaded by riparian forest dominated by *Dacryodes excelsa* Vahl. (tabonuco) and *Prestoea montana* (R. Graham) Nichols. (sierra palm). Litter fall is continuous throughout the year, with maxima and minima occurring between April–June and December–March, respectively (Zou *et al.*, 1995). Stream temperature is similar in both streams and ranges from 18 to 26 °C (mean: 22 °C; A. Ramírez, unpubl. data). Discharge responds rapidly to local storm events, is highly variable and shows no major seasonal pattern.

Table 1 Long-term mean discharge and nutrient chemistry data (±1 SE) for the Prieta and Bisley streams, Luquillo Experimental Forest, Puerto Rico (January 2000–October 2003)

Variable	Prieta	Bisley
Discharge (L s^{-1})	26	24
NH_4-N (μM)	0.5 ± 0.03	0.3 ± 0.04
NO_3-N (μM)	4.4 ± 0.19	7.9 ± 0.31
TDN (μM)	9.6 ± 0.50	12.6 ± 0.29
SRP (μM)	0.3 ± 0.02	0.65 ± 0.02

TDN, total dissolved nitrogen; SRP, soluble reactive phosphorus.

Stream communities of El Yunque contain up to 10 species of shrimps, including four species of Atyidae (*Atya lanipes* Holthuis, *Atya innocuous* [Herbst], *Atya scabra* [Leach], and *Micratya poeyi* [Guérin-Méneville]), one species of Xiphocarididae (*Xiphocaris elongata* [Guérin-Méneville]) and five species of Palaemonidae (*Macrobrachium carcinus* [L.], *Macrobrachium faustinum* [De Saussure], *Macrobrachium crenulatum* Holthuis, *Macrobrachium acanthurus* [Wiegmann], and *Macrobrachium heterochirus* [Wiegmann]). The shrimp assemblage in the Prieta is dominated by *X. elongata* and *A. lanipes* (>90% of individuals), with lower numbers of *A. innocuous*, *A. scabra*, *Micratya poeyi*, and *Macrobrachium* spp. The Bisley contains primarily *X. elongata*, *M. carcinus* and *M. faustinum* (Covich & McDowell, 1996). The two streams also differ with respect to densities of both shrimps and fishes. Our study reach on the Prieta is just upstream of a >10-m waterfall that excludes all fishes except the algivorous goby *Sicydium plumieri* (Bloch). The absence of the predatory mountain mullet (*Agonostomus monticola* [Bancroft]) and American eel (*Anguilla rostrata* [Lesueur]) results in relatively high densities of shrimps in this reach (up to 25 individuals m^{-2}; Covich, 1988a; Covich *et al.*, 2009). Our study reach on the Bisley has no waterfall downstream, supports populations of *Agonostomus* and *Anguilla*, and consequently has densities of shrimps that are two orders of magnitude lower than those in the Prieta (*c.* 0.1 individuals m^{-2}, mostly *X. elongata*; Townsend *et al.*, 2002; Covich *et al.*, 2009).

Shrimp excretion and body content measurements

Shrimp excretion rates were measured during two periods in each stream: 23–26 March and 24 November–20 December 2004. Shrimps were captured using dip and seine nets. To aid capture, we sometimes used short (<1 s) pulses from a backpack electrofisher to dislodge shrimps into the current where they could be more easily seined. Shrimps were never incapacitated during capture using this method. Shrimps were held for <15 min before excretion incubations began. Incubations were carried out by introducing single shrimps into 100 mL of filtered (glass-fibre, 0.7-μm pore size) stream water held in polyethylene bags (22 × 12 cm) suspended in the stream. Timed incubations lasted *c.* 60 min (range 49–100 min). At the start and conclusion of the incubation, two 20-mL water samples were collected and filtered (0.7-μm pore size). The water samples and shrimp from each incubation were then placed on ice and frozen within 6 h.

Water samples were analysed for ammonium, total dissolved nitrogen (TDN), and total dissolved phosphorus (TDP). Ammonium was measured using the phenol-hypochlorite colorimetric method (Solorzano, 1969) automated with a SmartChem robotic analyzer (Westco Scientific Instruments, Inc., Brookfield, CT, U.S.A.). TDN was measured using a Shimadzu TOC 5000 carbon analyzer (Shimadzu Corp., Columbia, MD, U.S.A.) connected to an Antek 720C chemiluminescent nitrogen detector (Antek Instruments, Houston, TX, U.S.A.; Merriam, McDowell & Currie, 1996). TDP was measured using the molybdate blue-ascorbic acid reaction after digestion by acid-persulphate oxidation (APHA, 1998). Shrimps were thawed, measured (ocular carapace length, mm), dried at 60 °C for 3 days, weighed (±0.1 mg) and ground by hand using a pestle and mortar. Ground subsamples were subsequently analysed for per cent C and N content on a Perkin Elmer Model 2400 Series II CHN analyzer (Perkin Elmer, Waltham, MA, U.S.A.). Per cent P content of ground subsamples (*c.* 0.5 mg) was measured in 10 mL of deionized water using the same methods as TDP mentioned earlier. Ground bovine liver (NIST 1577a; 1.11% P) was used as an organic P standard in all digestions. Excretion rates of ammonium, TDN and TDP were expressed as μmol shrimp^{-1} h^{-1} and plotted against body size (dry mass, g).

Basal resource stoichiometry

We collected samples of dominant basal resources from each stream for C, N and P analysis during March and November–December 2004. Microbially conditioned, mixed-species leaf litter was collected by hand along each stream reach. Suspended fine particulate organic matter was collected by filtering *c.* 4 L of stream water through 0.7-μm glass-fibre filters (n = 5). Periphyton was collected by brushing cobbles (n = 5) and filtering slurries onto glass-fibre filters. Basal resource samples were analysed for C, N and P content using the same methods as earlier.

Estimating shrimp biomass and whole-reach excretion

To estimate abundance and biomass of shrimps in the Prieta, we used pass-depletion sampling on two dates (20 February and 1 August 2005). On each date, six sub-reaches (three pools and three riffles) were sampled based on feasibility of depletion, which required that the upstream and downstream ends be effectively blocked with seine nets (4-mm mesh). After blocking both ends of a sub-reach, a third seine net was dragged in a downstream direction by two people so that shrimps were captured in the downstream net. The downstream net was briefly removed, captured shrimp were placed in a bucket with stream water, and the net was replaced. This process was repeated 3–5 times in each sub-reach, and captured shrimp from each run were temporarily held in separate buckets. All shrimps were identified, counted, measured (orbital carapace length) with callipers, and returned to the stream. Total abundance was estimated using maximum-likelihood methods (Removal Sampling 1.2, Pisces Conservation Ltd., U.K.), and size-class proportions of captured shrimps were applied to bootstrapped total abundance estimates. Biomass of individuals was calculated using orbital carapace length-dry mass regressions. Depletion sampling was attempted in the Bisley, but shrimp abundance was too low for this method to be effective. Consequently, we used abundance data generated from minnow-trap sampling in 2005 (Cross *et al.*, 2008).

The surface area of each sub-reach was measured to estimate shrimp abundance and biomass per square metre. We used a bootstrap-resampling technique to approximate the true distribution and variability of shrimp population abundance and biomass (Efron &

Tibshirani, 1993). For each genus on each sampling date, abundance data from the six sub-reaches were resampled with replacement to generate 1000 estimates of mean abundance. This process was repeated for each date, and the values for multiple dates were averaged to get 1000 estimates of mean annual abundance. From these data, we calculated a grand mean with bootstrapped 95% confidence intervals.

We combined shrimp density data with field measurements of excretion to calculate whole-reach estimates of nutrient excretion by the shrimp community in each stream. Whole-reach rates were calculated using the allometric excretion equations obtained in this study and by summing the proportional contributions of 1-mm (orbital carapace length) size-classes for each genus. If allometric relationships did not differ significantly between the two genera, we used the same equation for both. The same excretion equations were used in both the Prieta and Bisley streams. *Atya* were present at such low densities in the Bisley that we included only *Xiphocaris* in the whole-stream excretion calculations for that stream. The *Atya* equation for TDP excretion rate was used in the Prieta calculation even though the slope of the relationship was not significantly different from zero (see Results).

To shed light on temporal variability in the contribution of shrimp excretion to ambient nutrient pools, we adopted the approach of McIntyre *et al.* (2008) by calculating volumetric nutrient excretion and turnover distances. Volumetric excretion (E_V, mol nutrient per unit volume) was calculated as $E_V = (E_A \times A \times T)/V$, where E_A is areal excretion rate (mol nutrient m^{-2} h^{-1}), A is reach area (length × width, m^2), T is travel time through each reach (length/water velocity, h) and V is volume (length × cross-sectional area, m^3) at a given discharge. E_V is a useful metric because it describes the average addition of excreted nutrients to water as it flows along a given reach, assuming no uptake and perfect mixing (McIntyre *et al.*, 2008). Excretion turnover distance (m) is the distance required for excretion to turn over the ambient nutrient pool completely and was calculated by dividing ambient nutrient concentration (M) by E_V (M) and multiplying by the reach length (m) for which E_V was calculated (100 m). We calculated volumetric excretion and turnover distance of NH_4-N, TDN and TDP in both streams at long-term mean discharge and ambient nutrient concentrations (see Table 1). Given the flashy hydrology of our study sites, we also estimated temporal variability in NH_4-N turnover distance in the Prieta using our estimates of shrimp density and NH_4-N excretion rates, a 3-year record of weekly discharge and ambient NH_4-N concentration data, and relationships among discharge, reach depth and width. Area was kept constant in the numerator of the equation in these long-term calculations (i.e. shrimp densities declined in proportion to increasing discharge and reach area, but were otherwise not affected by changes in discharge). NH_4-N chemistry and discharge data were collected from the Prieta weekly between February 2000 and October 2003 as part of the Luquillo LTER sampling program using analytical techniques described earlier for NH_4-N.

Nutrient-uptake rates and long-term nutrient chemistry

Ammonium and phosphorus uptake rates were measured simultaneously using a single short-term nutrient release in each stream during February 2006 (Webster & Valett, 2006). Ammonium-N was added as NH_4Cl (target concentration 2.1 μM). Phosphorus was added as KH_2PO_4 (target concentration 1.6 μM). Chloride was used as a conservative tracer for calculation of discharge and dilution along each reach. Water samples were analysed as described earlier. Three caveats must be considered with regard to the results of our nutrient releases. First, a single release in each stream precludes any estimate of temporal variability in uptake kinetics. Second, the enrichment method we used may have overestimated uptake lengths because of saturation of uptake (Mulholland *et al.*, 2002). Third, simultaneous addition of nitrogen and phosphorus may have relaxed limitation of a primarily limiting nutrient, potentially altering demand for the other nutrient. While these caveats limit strong inference from our results, we chose to make this comparison of areal excretion rates with areal uptake rates because it is the most meaningful measure of ecosystem-level significance of consumer excretion.

Statistical analysis

Our initial approach to analysing excretion and body content data was to use multiple analysis of covariance (MANCOVA), with genus and stream as factors and body mass as the covariate. We combined data

from the two sampling periods to simplify statistical analyses because we were less interested in potential temporal differences in allometric excretion and body content relationships. Dependent variables in the excretion MANCOVA were excretion rates of ammonium, TDN and TDP (μmol shrimp^{-1} h^{-1}) and TDN:TDP (hereafter N:P) excretion ratio. Dependent variables in the body content MANCOVA were %C, %N, %P, C:N, N:P, and C:P. Preliminary analyses showed that the genus × stream interaction term was not significant in either the excretion or body content MANCOVA, so we repeated both MANCOVAS without the stream factor. MANCOVA significance indicated that one or more of the dependent variables differed between genera (Wilks's λ, $P < 0.05$) and was followed by individual one-way analysis of covariance (ANCOVA) for each dependent variable, with genus as the factor and body mass as the covariate. Significant differences between slopes or intercepts were followed by individual regressions for each genus. Per cent data were arcsine square-root transformed and all other data were log transformed to meet assumptions of normality and equal variance.

Per cent C, N and P (dry mass) and nutrient ratios of epilithon and seston were initially analysed using multiple analysis of variance (MANOVA) with resource type and stream as factors. MANOVA significance indicated that one or more of the dependent variables differed between factors (Wilks's λ, $P < 0.05$) and was followed by individual one-way analysis of variance (ANOVA) for each dependent variable, with resource type and stream as the factors. Significant differences among factors were followed by Tukey Honestly Significant Difference (HSD) tests. Per cent data were arcsine square-root transformed and ratios were log transformed to meet assumptions of normality and equal variance. Data for leaf litter were excluded from statistical analyses because of the low number of these composite samples.

Results

Shrimp body content stoichiometry

MANCOVA results indicated that there were significant differences in nutrient content (%C, N and P) and ratios (C:N, N:P and C:P) with body size and between shrimp genera (Wilks's λ, $P < 0.0001$). Per cent C, N and P of *Xiphocaris* were all significantly higher than those of *Atya* ($P < 0.0001$ for all three relationships; see Fig. 1a–c). Body content C:N of *Atya* was significantly higher than that of *Xiphocaris* ($P < 0.001$; Fig. 1d). Body content N:P of *Xiphocaris* was significantly higher than that of *Atya* ($P < 0.016$). The two genera did not differ significantly with respect to body content C:P (Fig. 1f).

Relationships of elemental content with body mass were significant only for %P (*Xiphocaris*: $P = 0.03$; *Atya*: $P = 0.0006$); slopes of this relationship did not differ between the two genera ($P = 0.65$ for the interaction term; see Fig. 1c). Relationships of C:N with body mass were significant only for *Atya* ($P = 0.01$; Fig. 1d). Relationships of N:P with body mass were weakly significant for *Atya* ($P = 0.07$; Fig. 1e). The overall relationship of C:P with body mass was significant and positive for both taxa ($P < 0.0001$; Fig. 1f).

Basal resource stoichiometry

MANOVA results indicated that there were significant differences in nutrient content (%C, N and P) and ratios (C:N, N:P and C:P) between resource types (epilithon and seston) and streams (Wilks's λ, $P < 0.0001$). Patterns between %C and %N of epilithon and seston in the two streams were similar, with significantly higher content of both elements in epilithon from the Prieta (Fig. 2a,b). Per cent P of epilithon was significantly higher in the Prieta, while seston did not differ between the two streams (Fig. 2c). No differences in C:N were found between resources or streams (Fig. 2d). The two streams did not differ in epilithon N:P, but seston N:P was significantly higher in the Prieta (Fig. 2e). Epilithon C:P was significantly higher in the Prieta; seston N:P did not differ between the streams (Fig. 2f).

Differences in elemental composition of leaf litter between the two streams were not analysed statistically. Per cent C, C:N and C:P of leaf litter were *c.* 4 times higher than those of epilithon and seston (Fig. 2a,d,f). Per cent N, %P and N:P of leaf litter were intermediate relative to those of epilithon and seston in either stream.

Shrimp excretion rates and ratios

Rates of NH_4-N and TDN excretion did not differ between the two shrimp genera (Fig. 3a,b), although the overall relationships with body mass were highly

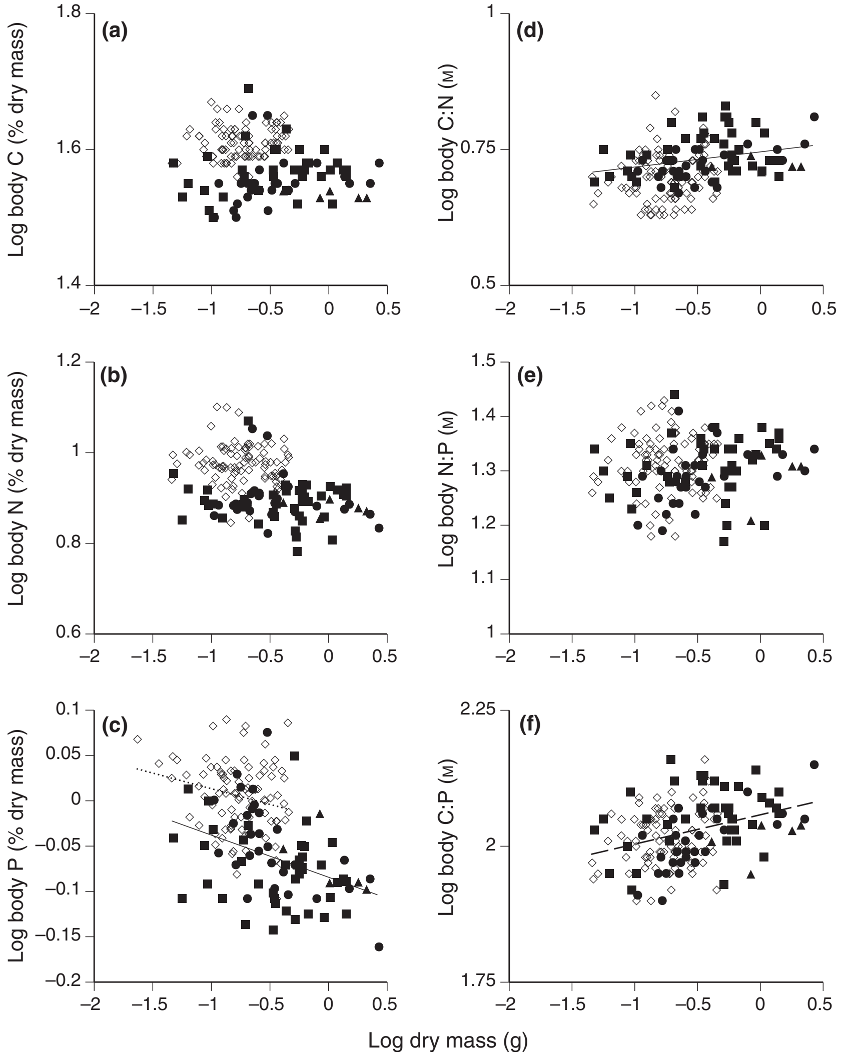

Fig. 1 Relationships between body size and body elemental stoichiometry in two genera of freshwater shrimps collected from two streams in northeastern Puerto Rico. Open diamonds = *Xiphocaris elongata*, filled symbols = *Atya* spp. (squares = *A. lanipes*, circles = *A. innocous*, triangles = *A. scabra*). Only significant genus-specific regressions ($P < 0.05$) are shown (dotted line = *Xiphocaris*, solid line = *Atya*); where regressions did not differ significantly between genera, a single dashed line is shown: (a) % carbon; (b) % nitrogen; (c) % phosphorus [*Atya*, $r^2 = 0.16$, log body %P = −0.046(log dry mass) − 0.084; *Xiphocaris*, $r^2 = 0.05$, log body %P = −0.035(log dry mass) − 0.021]; (d) molar carbon: nitrogen ratio [*Atya*, $r^2 = 0.09$, log body C:N = 0.028(log dry mass) + 0.75]; (e) molar nitrogen: phosphorus ratio; (f) molar carbon: phosphorus ratio [Taxa combined, $r^2 = 0.12$, log body C:P = 0.054(log dry mass) − 2.06]. Data from the two streams did not differ significantly and were combined.

significant ($P < 0.0001$ for both relationships). Patterns of TDP excretion between the two genera were more complex, with significant differences in both the intercept ($P < 0.0001$) and slope ($P < 0.006$) of the relationship with body mass (Fig. 3c). Relationships of TDP excretion rate with body mass were only significant for *Xiphocaris* ($P < 0.0001$). *Xiphocaris* had higher TDP excretion rates than *Atya* at most body masses measured. Excretion N:P ratio differed significantly between the two genera ($P < 0.0001$) and showed contrasting patterns with body mass (Fig. 3d). Excretion N:P increased significantly with body mass in *Atya* ($P = 0.02$), while it declined in *Xiphocaris* ($P = 0.05$). This difference in N:P excretion allometry was driven by relatively steeper increases in P excretion with body mass in *Xiphocaris*, giving rise to a negative excretion N:P slope.

When examined across both taxa, relationships between excretion rates and body content stoichiometry were statistically highly significant, but had weak explanatory power (NH_4-N excretion rate versus %N body content, $P = 0.0008$, $r^2 = 0.07$; TDN excretion rate versus %N body content, $P = 0.0007$, $r^2 = 0.07$; TDP excretion rate versus %P body content, $P = 0.04$, $r^2 = 0.03$; data not shown). Slopes for nitrogen were negative, while that for phosphorus was weakly positive. The relationship between excretion N:P and body content N:P across both taxa was also weak ($P = 0.06$, $r^2 = 0.02$; data not shown).

Whole-stream shrimp excretion estimates

Mean density of *Atya* was 14.5 m^{-2} in the Prieta and was too low to quantify in the Bisley (see Table 2 for

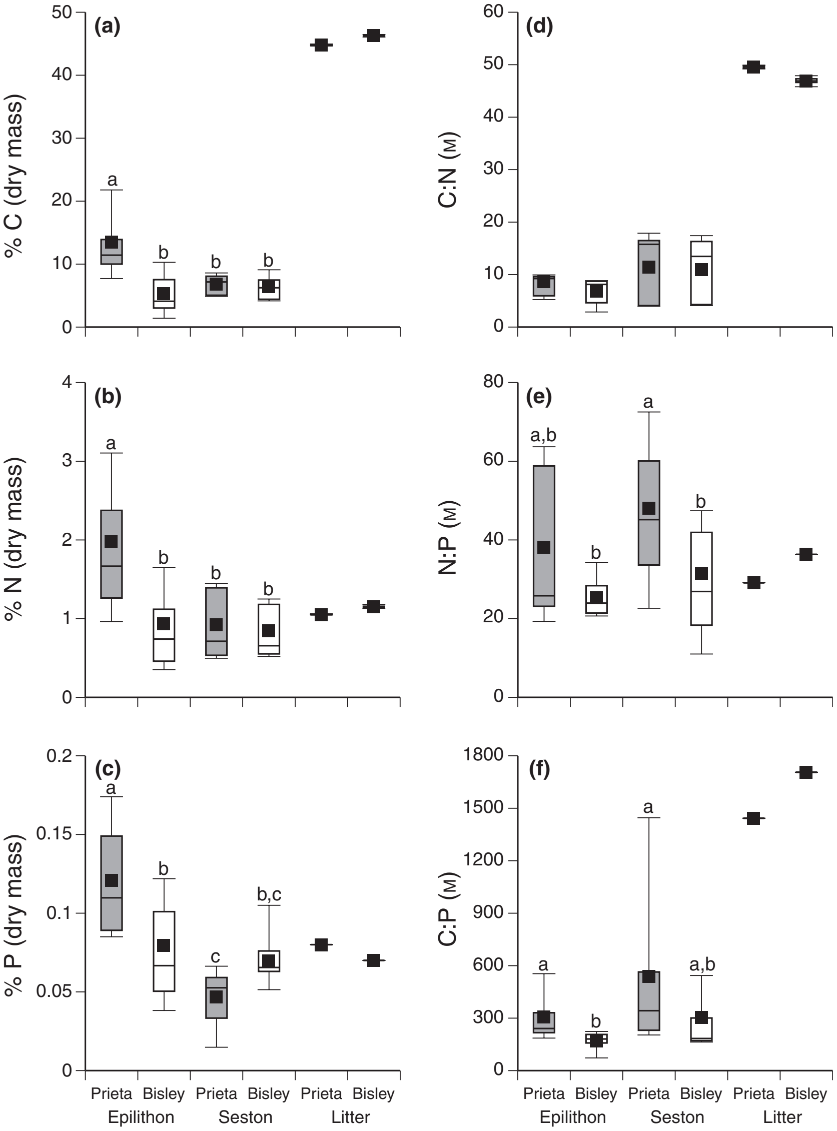

Fig. 2 Box plots of food resource (epilithon, seston and leaf litter) stoichiometry in the two study streams in northeastern Puerto Rico: (a) % carbon, (b) % nitrogen, (c) % phosphorus, (d) molar carbon: nitrogen ratio, (e) molar nitrogen: phosphorus ratio, (f) molar carbon: phosphorus ratio. The top, bottom, and line through the middle of the box correspond to the 75th, 25th, and 50th percentile (median), respectively. The whiskers extend from the 10th percentile to the 90th percentile. The solid square represents the arithmetic mean. Different letters above bars indicate significant differences ($P < 0.05$). Leaf litter was excluded from statistical analyses.

95% confidence intervals derived from bootstrapping of density data). *Atya* biomass in the Prieta was 5.2 g DM m^{-2}. Abundance and biomass of *Xiphocaris* in the Prieta was 13.2 m^{-2} and 2.6 g DM m^{-2}, respectively. Abundance and biomass of *Xiphocaris* in the Bisley were more than a hundredfold lower than that measured in the Prieta (0.12 m^{-2} and 0.09 g DM m^{-2}; Table 2). Combining density data, size-class distributions, and allometric excretion relationships yielded whole-stream (i.e. both genera) excretion estimates of 22.3 μmol m^{-2} h^{-1} and 37.5 μmol m^{-2} h^{-1} for NH_4-N and TDN, respectively, in the Prieta (Table 2). Whole-stream TDP excretion in the Prieta was much lower (1.1 μmol m^{-2} h^{-1}; Table 2). Low shrimp densities in the Bisley resulted in extremely low whole-stream excretion estimates, for both nitrogen (0.2 μmol m^{-2} h^{-1} NH_4-N and 0.26 μmol m^{-2} h^{-1} TDN) and phosphorus (0.015 μmol m^{-2} h^{-1}; Table 2).

Whole-stream shrimp excretion rates were compared with whole-stream nutrient uptake measured on a single date (see Table 3). In the Prieta, excretion rate of NH_4-N was equivalent to 21% of NH_4-N uptake measured during the single release (Table 4). Excretion by shrimp could potentially meet 5% of P demand in the Prieta. Equivalent proportions of NH_4-N and P demand excreted by shrimp in the Bisley were 0.5% and <0.1%, respectively (Table 4).

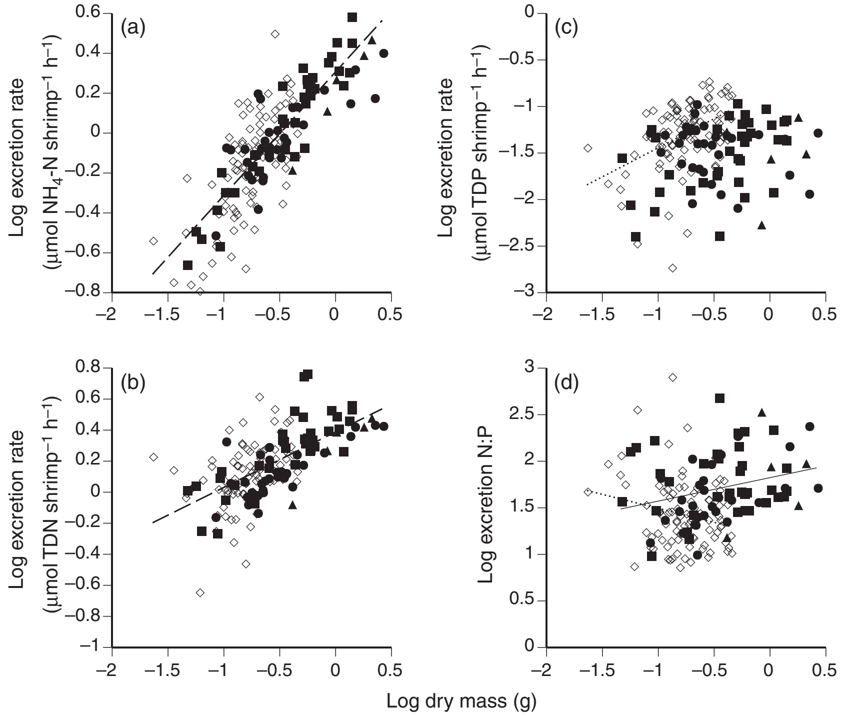

Fig. 3 Relationships between body size and individual nutrient excretion rates and N:P ratio in two genera of freshwater shrimps collected from two streams in northeastern Puerto Rico. Open diamonds = *Xiphocaris elongata*, filled symbols = *Atya* spp. (squares = *A. lanipes*, circles = *A. innocous*, triangles = *A. scabra*). Only significant genus-specific regressions ($P < 0.05$) are shown (dotted line = *Xiphocaris*, solid line = *Atya*); where regressions did not differ significantly between genera, a single dashed line is shown: (a) NH_4-N excretion [Taxa combined, $r^2 = 0.66$, log NH_4-N excretion rate = 0.62(log dry mass) + 0.31]; (b) total dissolved nitrogen excretion [Taxa combined, $r^2 = 0.39$, log TDN excretion rate = 0.36(log dry mass) + 0.39]; (c) total dissolved phosphorus excretion [*Xiphocaris*, $r^2 = 0.20$, log TDP excretion rate = 0.62(log dry mass) − 0.82]; (d) molar total dissolved nitrogen: total dissolved phosphorus ratio [*Atya*, $r^2 = 0.08$, log excretion N:P = 0.25(log dry mass) + 1.82; *Xiphocaris*, $r^2 = 0.04$, log excretion N:P = −0.30(log dry mass) − 1.19]. Data from the two streams did not differ significantly and were combined.

Volumetric excretion of NH_4-N, TDN and TDP in the 100-m reach of the Prieta amounted to 63, 106 and 3 nM, respectively (see Table 4 for 95% confidence intervals derived from bootstrapping of density data). Equivalent estimates for the low-shrimp Bisley were 1, 1 and 0 nM for NH_4-N, TDN and TDP, respectively (Table 4). Combined with ambient nutrient concentrations, these volumetric excretion estimates resulted in excretion turnover distances in the Prieta of 786 m for NH_4-N and *c.* 9 km for TDN and TDP (Table 4). Excretion turnover distances were >20 km for all three nutrients in the Bisley. Long-term modelling revealed considerable variability in the distance required for shrimp excretion to turn over the ambient NH_4-N pool in the Prieta (Fig. 4a; range 12–8740 m, mean 661 m). This variability was driven by large ranges in both discharge and ambient NH_4-N concentration. Most calculated distances fell between 200 and 600 m (Fig. 4b).

Discussion

The relative magnitude and ecosystem-level significance of consumer excretion depend on a combination of relatively well-understood biotic and abiotic factors (Schaus *et al.*, 1997; Vanni *et al.*, 2002; Hall *et al.*, 2007; McIntyre *et al.*, 2008). These factors include those related to supply rate from consumers (i.e. biomass, consumer elemental demand and diet stoichiometry), those related to total pools and turnover of elements (i.e. habitat volume, turnover rate and the ambient concentration of limiting nutrients) and those related to nutrient sinks (i.e. biomass, activity and stoichiometry of microbes and primary producers). The relative contributions of different factors in controlling the potential significance of excretion have rarely been studied, however (but see McIntyre *et al.*, 2008). We used measurements of shrimp excretion rates, densities, and long-term records of discharge and nutrient

© 2010 Blackwell Publishing Ltd, *Freshwater Biology*, **55**, 2047–2061

Variable	Prieta	Bisley
Shrimp abundance		
Atya		
Abundance (m^{-2})	14.5 (7.0–22.8)	–
Biomass (g DM m^{-2})	5.2 (2.5–8.3)	–
Xiphocaris		
Abundance (m^{-2})	13.2 (8.8–18.0)	0.12 (0.02–0.23)
Biomass (g DM m^{-2})	2.6 (1.7–3.6)	0.09 (0.01–0.17)
Whole-stream shrimp excretion		
Atya		
NH_4-N excretion (μmol m^{-2} h^{-1})	12.8 (6.2–20.2)	–
TDN excretion (μmol m^{-2} h^{-1})	20.0 (9.6–31.4)	–
TDP excretion (μmol m^{-2} h^{-1})	0.40 (0.20–0.64)	–
Xiphocaris		
NH_4-N excretion (μmol m^{-2} h^{-1})	9.5 (6.3–12.9)	0.20 (0.03–0.38)
TDN excretion (μmol m^{-2} h^{-1})	17.5 (11.7–23.9)	0.26 (0.04–0.49)
TDP excretion (μmol m^{-2} h^{-1})	0.71 (0.47–0.96)	0.015 (0.002–0.028)
Total excretion		
NH_4-N excretion (μmol m^{-2} h^{-1})	22.3 (12.5–33.1)	0.20 (0.03–0.38)
TDN excretion (μmol m^{-2} h^{-1})	37.5 (21.3–55.3)	0.26 (0.04–0.49)
TDP excretion (μmol m^{-2} h^{-1})	1.1 (0.7–1.6)	0.015 (0.002–0.028)

TDN, total dissolved nitrogen; TDP, total dissolved phosphorus.

Table 2 Densities of *Atya* and *Xiphocaris* shrimps and whole-stream nutrient excretion estimates for the Prieta and Bisley streams, Luquillo Experimental Forest, Puerto Rico. Abundances were estimated in February and August 2005. Excretion was measured in March and November–December 2004. Values are means with 95% confidence intervals based on bootstrapping of abundance estimates

Variable	Prieta	Bisley
Discharge (L s^{-1})	26	25
NH_4-N		
Ambient concentration (μM)	0.24	0.18
Uptake length (m)	79 (63–104)	141 (133–150)
Uptake rate (μmol m^{-2} h^{-1})	105.3 (80.0–132.0)	39.3 (36.9–41.7)
Uptake velocity ($\times 10^{-4}$ m s^{-1})	1.24 (0.94–1.56)	0.61 (0.58–0.65)
SRP		
Ambient concentration (μM)	0.18	0.37
Uptake length (m)	270 (157–959)	417 (286–767)
Uptake rate (μmol m^{-2} h^{-1})	23.7 (6.7–40.7)	27.9 (15.2–40.7)
Uptake velocity ($\times 10^{-4}$ m s^{-1})	0.36 (0.10–0.63)	0.21 (0.11–0.30)

Table 3 Discharge values and uptake parameters for NH_4-N and P during the short-term releases conducted during February 2006 in the Prieta and Bisley streams, Luquillo Experimental Forest, Puerto Rico. Error estimates for uptake parameters (in parentheses) are derived from the standard error for the slope of dilution-corrected nutrient concentration regressed against distance

concentration to estimate spatial and temporal variability in the relative magnitude of excretion by tropical freshwater shrimps. Our results demonstrate the combined effects of biotic and abiotic factors in controlling the significance of consumer excretion for nutrient cycling in aquatic ecosystems. Body content stoichiometry affected excretion rates and ratios of shrimps only weakly. Differences in community-level excretion were related to variation in shrimp biomass between streams, dictated by stream geomorphology and the resulting presence of predatory fishes. Temporal variability in the contribution of shrimp excretion to ambient nutrient pools was driven by temporal patterns in stream discharge and nutrient concentrations, which affected the magnitude of nutrients excreted by shrimps relative to the ambient pool.

Stoichiometry of shrimp body content and excretion

Growth rate may drive some of the differences we observed in body content stoichiometry (e.g. %P), both between the two shrimp genera and with increasing body mass. Growth rate and P associated with RNA are expected to decline with increasing body mass (Elser *et al.*, 2003). Moreover, *Xiphocaris* exhibits faster growth rates than *Atya*, at least at smaller body sizes (Cross *et al.*, 2008), which may partly explain its higher per cent P body content.

Table 4 Excretion rates of shrimp communities relative to uptake rates measured in February 2006 in the Prieta and Bisley streams, Luquillo Experimental Forest, Puerto Rico. Volumetric rates are also shown, as are the distances required for shrimp community excretion to turn over the ambient nutrient pools (both based on long-term mean discharge and nutrient concentrations; see Table 1). Values are means with 95% confidence intervals based on bootstrapping of abundance estimates

Variable	Prieta	Bisley
Excretion relative to uptake		
NH_4-N uptake (%)	21 (12–31)	0.5 (0–1)
P uptake (%)	5 (3–7)	<0.1
Volumetric excretion		
NH_4-N (nM)	64 (36–94)	1 (0–1)
TDN (nM)	107 (61–158)	1 (0–2)
TDP (nM)	3 (2–5)	0.0
Turnover distance		
NH_4-N (m)	786 (530–1402)	>20000
TDN (m)	8974 (6086–15800)	>20000
TDP (m)	9561 (6573–15024)	>20000

TDN, total dissolved nitrogen; TDP, total dissolved phosphorus.

Other differences in body stoichiometry between the two genera were probably a result of differing structural composition. For example, *Atya* has a relatively more calcified exoskeleton than *Xiphocaris* and a consequently higher ash content (W.F. Cross, unpubl. data), potentially explaining higher %C and %N in *Xiphocaris*.

Differences in excretion rates and ratios between genera and body sizes were only weakly related to body content stoichiometry. In contrast, Vanni *et al.* (2002) reported strong relationships between N and P excretion rates and body content in 13 families of fish and amphibians. However, this broader-scale study encompassed far greater ranges in body content stoichiometry than are reported here (e.g. <1 to >4%P versus 0.7–1.2%P; N:P of *c.* 5 to >22 versus 15–28). Consideration of many of the families included in the Vanni *et al.* (2002) study in isolation likely would have failed to show such strong

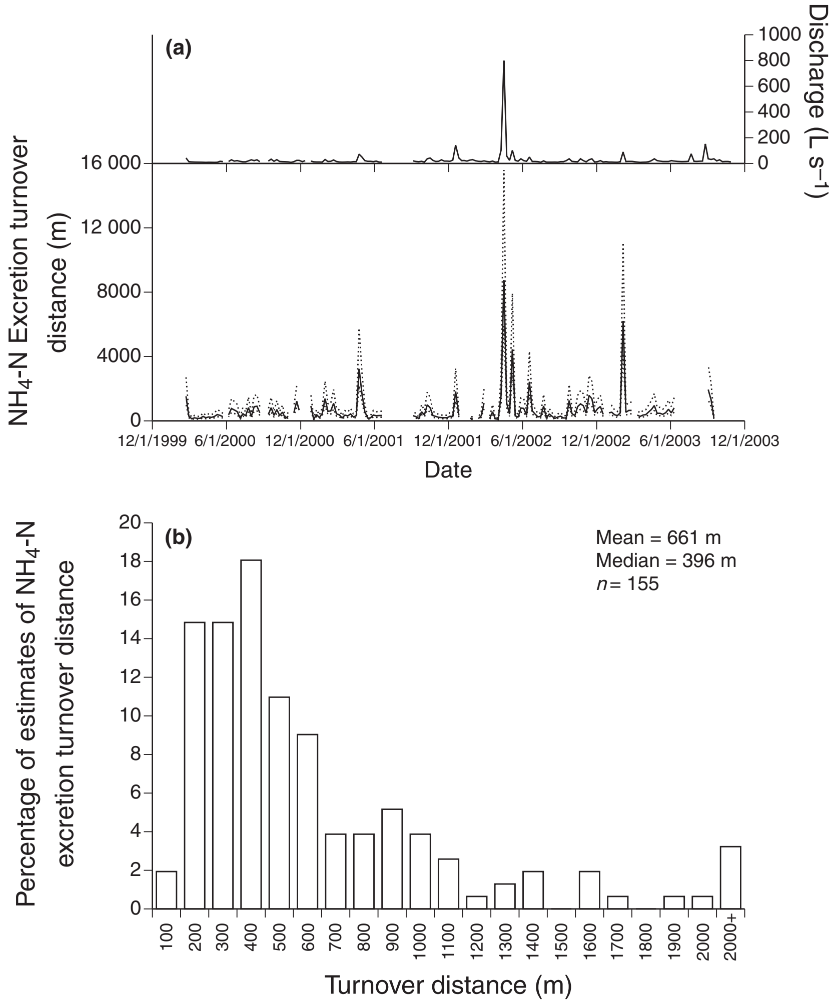

Fig. 4 (a) Discharge and estimated NH_4-N excretion turnover distance (distance required for shrimp excretion to turn over the ambient NH_4-N pool) based on changes in discharge and ambient NH_4-N in the Prieta stream from February 2000 to October 2003. Shrimp densities were based on sampling during 2005. Solid line represents the mean turnover distance ±95% confidence intervals (dashed lines) based on bootstrapping of shrimp density data (see text). Gaps indicate missing data. (b) frequency histogram of NH_4-N turnover distance estimates in the Prieta stream from February 2000 to October 2003.

relationships between the stoichiometry of excretion and body content. By including only two taxa in our study, we may have lacked the broad range in body content stoichiometry needed to isolate its effects on excretion rates and ratios.

Consequently, much of the unexplained variance in excretion rates and ratios with body mass or body content stoichiometry was probably driven by variability in diet (or proportions of diet components). Based on body stoichiometry alone, *Xiphocaris* would be expected to excrete both N and P at lower rates than *Atya*, given its higher per cent content of both elements (Sterner & Elser, 2002). In fact, excretion rate of N in *Xiphocaris* was statistically indistinguishable from that of *Atya*, while its rate of P excretion was higher at most body sizes. Consideration of stoichiometric balance therefore suggests that the diets of these two taxa differ fundamentally in N and P content, with *Xiphocaris* consuming organic matter of higher content in both elements. This is perhaps not surprising, given that *Xiphocaris* uses tiny pincer-like chelipeds to shred leaf litter, collect fine particles or periphyton, and capture insect prey. In contrast, *Atya* have cheliped fans used to feed less selectively by filtering seston or scrubbing fine benthic particles (Covich, 1988a). Although few dietary comparisons have been made between *Atya* and *Xiphocaris*, stable isotope evidence from stream reaches near our study sites suggests that *Xiphocaris* consumes relatively more algae (March & Pringle, 2003).

Shrimp effects on basal resource stoichiometry

Our two study sites differed greatly in shrimp community structure and biomass as a result of a >10-m waterfall downstream from our study reach on the Prieta, which excludes important fish predators of *Xiphocaris* and *Atya* that are present in the Bisley reach. The consequences of this predator-mediated effect on shrimp biomass have been reported for some parameters (e.g. differences in benthic organic matter quantity and quality; Pringle *et al.*, 1999; March *et al.*, 2002). In this study, we observed differences in the stoichiometry of some basal resources that are consistent with previous observations. In particular, C, N and P content of epilithon was higher in the Prieta (high shrimp biomass), indicative of the lower inorganic content shown to result from shrimp feeding and bioturbation (Pringle *et al.*, 1999). Any differences in resource stoichiometry between the two streams were not reflected in excretion rates, which did not differ between our two study sites. Lack of differences between the two streams in excretion rates (despite differences in resource stoichiometry) may have been because of discrepancy between measured nutrient content of bulk resources and that of food actually ingested.

Although we found no evidence for shrimp-mediated changes to food resources resulting in shifts in excretion rates or ratios, the effects of shrimp feeding and bioturbation on organic matter storage, composition and activity suggest the potential for shrimps to have indirect effects on nutrient cycling (Vanni, 2002). For example, if shrimps clear sediment and maintain metabolically more active biofilms through bioturbation (Pringle *et al.*, 1999; March *et al.*, 2002), N and P uptake might be expected to be higher in streams with higher shrimp biomass. Our single nutrient releases followed this pattern, with uptake velocities for both N and P being two times higher at the high shrimp-biomass Prieta site. Consequently, the indirect effects of shrimp feeding and activity probably interact with the direct effects of shrimps (those mediated via excretion) at high shrimp densities. However, whether direct or indirect effects of consumption have greater effects on nutrient cycling is an important question that deserves more attention in this and other study systems (e.g. Knoll *et al.*, 2009).

Ecosystem-level significance of excretion

We assessed the ecosystem-level significance of shrimp excretion by comparing shrimp community-level excretion with estimates of areal N and P uptake obtained from nutrient releases. We conducted single nutrient releases in each stream, precluding any assessment of temporal or concentration-dependent variability in nutrient uptake. Our estimate of NH_4-N uptake velocity in the Bisley was lower than those obtained in an earlier study that used a 42-d tracer-level release of $^{15}NH_4$ in the same stream reach (1.46–1.80×10^{-4} m s^{-1}; Merriam *et al.*, 2002), suggesting that we underestimated areal uptake rates. By using a mean uptake velocity (1.648×10^{-4} m s^{-1}) from Merriam *et al.* (2002) and the ambient NH_4-N concentration during the Bisley release (0.18 μM), we obtain an areal uptake rate of 107 μmol m^{-2} h^{-1} for the Bisley. This estimate is about 2.5 times higher than that

measured during our NH_4-N release. We cannot assume the same nutrient kinetics (and, therefore, discrepancy in uptake rate estimates) in the high-shrimp Prieta, but we likely also underestimated uptake rate in this stream. Community-level NH_4-N excretion by shrimps in the high-biomass stream (Prieta) was equivalent to approximately one-fifth of the stream's NH_4-N uptake measured using the nutrient release. Importantly, this contribution would decrease in proportion to any increase in areal uptake rate. We stress, therefore, that our estimate of excretion representing 20% of NH_4-N uptake (and of NH_4-N regeneration, assuming steady state conditions) must be considered a maximum estimate of the contribution of excretion. With regard to P, the equivalent proportion of shrimp excretion to P uptake was much lower in the Prieta, driven by low P excretion, while low-shrimp biomass in the Bisley made shrimp contributions insignificant for both N and P, even compared to the relatively lower uptake rates in this stream.

The contrasting patterns in shrimp biomass and potential contributions of excretion to nutrient uptake that we observed highlight spatial differences in the significance of consumer excretion to nutrient cycling that may be common to aquatic ecosystems. In our study system, natural waterfalls exclude fish predators, resulting in shifts in the relative abundance of consumer taxa. The potential for such shifts to increase excretion rate depends on whether excretion by predatory taxa can compensate for losses from reduced prey biomass. We have no data for biomass or excretion rates of predatory fishes at our low-shrimp site, but such compensation seems unlikely given their low abundance relative to that of shrimps in our high-shrimp stream (J.P. Benstead & W.F. Cross, pers. observ.). Even in high-shrimp streams, contributions from shrimp excretion are likely no more than 20% of uptake, underscoring the importance of relatively high consumer biomass in driving ecosystem-level significance of consumer excretion (see also Small, Helton & Kazanci, 2009).

We also assessed the potential for temporal variability in the importance of excretion by calculating distances required for excretion to turn over ambient nutrient pools under different conditions. This analysis highlighted the dominant role of abiotic factors in dictating temporal shifts in the significance of excretion: estimates of turnover distance were more sensitive to short-term changes in hydrology and ambient nutrient concentration than to relatively wide ranges in shrimp abundance (i.e. ±95% confidence intervals). Excretion turnover distance for NH_4-N ranged from 12 to 8740 m, while the mean range attributable to the 95% confidence intervals for shrimp biomass was 586 m. Excretion turnover distance is a recently introduced metric (McIntyre *et al.*, 2008), so few comparative data are available. In an N-limited tropical river, total NH_4-N excretion by a diverse community of fishes turned over the ambient NH_4-N pool over a distance of <300 m (McIntyre *et al.*, 2008). Fish biomass at this site was estimated at 44 g wet mass m^{-2}, compared to approximately 31 g wet mass m^{-2} of shrimps in our high-shrimp stream. Our estimates of NH_4-N excretion turnover distance are similar to those reported in this earlier study, despite lower consumer biomass and broad differences between the taxonomic composition of the consumer community involved. It must be noted, however, that we assumed constant shrimp biomass and excretion rates in our temporal analysis.

In conclusion, our results underscore the importance of biotic and abiotic factors that control consumer excretion and its significance for nutrient cycling in aquatic ecosystems. Although we found little evidence for a strong effect of body stoichiometry on excretion, the rates and ratios of excretion by individual shrimps were likely still controlled by interactions between resource stoichiometry and nutrient demands for growth and maintenance. Spatial differences in community-level excretion rates by shrimps were related to shrimp biomass, driven in turn by the presence of physical barriers that controlled the movement of predators of shrimp. Finally, abiotic factors had important effects on the contribution of consumer excretion, through temporal patterns in stream discharge and ambient nutrient concentrations. This combination of biotic and abiotic factors is not unique to our study system. Future studies that focus on the significance of consumer excretion in aquatic ecosystems will benefit from consideration of such factors, as well as the importance of excretion for nutrient cycling relative to the indirect effects of animals mediated by consumption (e.g. Knoll *et al.*, 2009).

Acknowledgments

This research was funded by the Luquillo LTER program (NSF DEB-0620919) in co-operation with the

Institute of Tropical Ecosystem Studies, University of Puerto Rico, and the International Institute of Tropical Forestry, Río Piedras, PR, and a Howard Hughes Medical Institute Undergraduate Science Program Education Grant (52002683) to Washington and Jefferson College. We are grateful to Elizabeth Reese for field assistance, to Jeff Merriam for conducting analyses of water and C:N samples and to Michael Chadwick for providing the bootstrapping spreadsheet for calculating shrimp densities. The manuscript benefited from comments by Michael Kendrick, James Ramsey, Ryan Sponseller and two anonymous reviewers. Finally, we thank Steve Thomas for useful discussions about assessment of the significance of consumer excretion in stream ecosystems.

References

American Public Health Association (APHA) (1998) *Standard Methods for the Examination of Water and Wastewater*, 20th edn. American Public Health Association, Washington, DC.

Covich A.P. (1988a) Atyid shrimp in the headwaters of the Luquillo Mountains, Puerto Rico: filter feeding in natural and artificial streams. *Verhandlungen Internationale Vereinigung für Theoretische und Angewandte Limnologie*, **23**, 2108–2113.

Covich A.P. (1988b) Geographical and historical comparisons of Neotropical streams: biotic diversity and detrital processing in highly variable habitats. *Journal of the North American Benthological Society*, **7**, 361–386.

Covich A.P. & McDowell W.H. (1996) The stream community. In: *The Food Web of a Tropical Rain Forest* (Eds D.P. Reagan & R.B. Waide), pp. 433–459. The University of Chicago Press, Chicago.

Covich A.P., Crowl T.A., Hein C.L., Townsend M.J. & McDowell W.H. (2009) Predator-prey interactions in river networks: comparing shrimp spatial refugia in two drainage basins. *Freshwater Biology*, **54**, 450–465.

Cross W.F., Benstead J.P., Frost P.C. & Thomas S.A. (2005) Ecological stoichiometry in freshwater benthic systems: recent progress and perspectives. *Freshwater Biology*, **50**, 1895–1912.

Cross W.F., Covich A.P., Crowl T.A., Benstead J.P. & Ramírez A. (2008) Secondary production, longevity and resource consumption rates of freshwater shrimps in two tropical streams with contrasting geomorphology and food web structure. *Freshwater Biology*, **53**, 2504–2519.

Efron B. & Tibshirani R. (1993) *An Introduction to the Bootstrap*. Chapman and Hall, London.

Elser J.J., Acharya K., Kyle M. *et al.* (2003) Growth rate – stoichiometry couplings in diverse biota. *Ecology Letters*, **6**, 936–943.

Findlay D.L., Vanni M.J., Paterson M., Mills K.H., Kasian S.E.M., Findlay W.J. & Salki A.G. (2005) Dynamics of a boreal lake ecosystem during a long-term manipulation of lake predators. *Ecosystems*, **8**, 603–618.

Hall R.O., Tank J.L. & Dybdahl M.F. (2003) Exotic snails dominate nitrogen and carbon cycling in a highly productive stream. *Frontiers in Ecology and the Environment*, **1**, 407–411.

Hall R.O., Koch B.J., Marshall M.C., Taylor B.W. & Tronstad L.M. (2007) How body size mediates the role of animals in nutrient cycling. In: *Body Size: The Structure and Function of Aquatic Ecosystems* (Eds A.G. Hildrew, D.G. Raffaelli & R. Edmonds-Brown), pp. 286–305. Cambridge University Press, Cambridge.

Heartsill-Scalley T., Scatena F.N., Estrada C., McDowell W.H. & Lugo A.E. (2007) Disturbance and long-term patterns of rainfall and throughfall nutrient fluxes in a subtropical wet forest in Puerto Rico. *Journal of Hydrology*, **333**, 472–485.

Kitchell J.F., O'Neill R.V., Webb D., Gallep G.W., Bartell S.M., Koonce J.F. & Ausmus B.S. (1979) Consumer regulation of nutrient cycling. *BioScience*, **29**, 28–34.

Knoll L.B., McIntyre P.B., Vanni M.J. & Flecker A.S. (2009) Feedbacks of consumer nutrient recycling on producer biomass and stoichiometry: separating direct and indirect effects. *Oikos*, **118**, 1732–1742.

March J.G. & Pringle C.M. (2003) Food web structure and basal resource utilization along a tropical island stream continuum, Puerto Rico. *Biotropica*, **35**, 84–93.

March J.G., Pringle C.M., Townsend M.J. & Wilson A.I. (2002) Effects of freshwater shrimp assemblages on benthic communities along an altitudinal gradient of a tropical island stream. *Freshwater Biology*, **47**, 377–390.

McIntyre P.B., Flecker A.S., Vanni M.J., Hood J.M., Taylor B.W. & Thomas S.A. (2008) Fish distributions and nutrient cycling in streams: can fish create biogeochemical hotspots? *Ecology*, **89**, 2335–2346.

Merriam J.L., McDowell W.H. & Currie W.S. (1996) A high-temperature catalytic oxidation technique for determining total dissolved nitrogen. *Soil Science Society of America Journal*, **60**, 1050–1055.

Merriam J.L., McDowell W.H., Tank J.L., Wollheim W.M., Crenshaw C.L. & Johnson S.L. (2002) Characterizing nitrogen dynamics, retention and transport in a tropical rainforest stream using an *in situ* ^{15}N addition. *Freshwater Biology*, **47**, 143–160.

Mulholland P.J. *et al.* (2002) Can uptake length in streams be determined by nutrient addition experiments?

Results from an interbiome comparison study. *Journal of the North American Benthological Society*, **21**, 544–560.

Pomeroy L.R., Matthews H.M. & Min H.S. (1963) Excretion of phosphate and soluble organic phosphorus compounds by zooplankton. *Limnology and Oceanography*, **8**, 50–55.

Pringle C.P., Hemphill N., McDowell W.H., Bednarek A. & March J.G. (1999) Linking species and ecosystems: different biotic assemblages cause interstream differences in organic matter. *Ecology*, **80**, 1860–1872.

Rigler F.H. (1961) The uptake and release of phosphorus by *Daphnia magna* Straus. *Limnology and Oceanography*, **6**, 165–174.

Schaus M.H., Vanni M.J., Wissing T.E., Bremigan M.T., Garvey J.E. & Stein R.A. (1997) Nitrogen and phosphorus excretion by detritivorous gizzard shad in a reservoir ecosystem. *Limnology and Oceanography*, **42**, 1386–1397.

Small G.E., Helton A.M. & Kazanci C. (2009) Can consumer stoichiometric regulation control nutrient spiraling in streams? *Journal of the North American Benthological Society*, **28**, 747–765.

Solorzano L. (1969) Determination of ammonia in natural waters by the phenolhypochlorite method. *Limnology and Oceanography*, **14**, 799–801.

Sterner R.W. & Elser J.J. (2002) *Ecological Stoichiometry*. Princeton University Press, Princeton.

Torres L.E. & Vanni M.J. (2007) Stoichiometry of nutrient excretion by fish: interspecific variation in a hypertrophic lake. *Oikos*, **116**, 259–270.

Townsend M.J., Crowl T.A., Phillips R., Covich A.P. & Scatena F.N. (2002) Indirect and direct abiotic controls on a species-poor stream insect assemblage. *Verhandlungen Internationale Vereinigung für Theoretische und Angewandte Limnologie*, **28**, 919–922.

Vanni M.J. (2002) Nutrient cycling by animals in freshwater ecosystems. *Annual Review of Ecology and Systematics*, **33**, 341–370.

Vanni M.J., Flecker A.S., Hood J.M. & Headworth J.L. (2002) Stoichiometry of nutrient recycling by vertebrates in a tropical stream: linking species identity and ecosystem processes. *Ecology Letters*, **5**, 285–293.

Webster J.R. & Valett H.M. (2006) Solute dynamics. In: *Methods in Stream Ecology* (Eds F.R. Hauer & G.A. Lamberti), pp. 169–185. Elsevier, Amsterdam.

Woodward G., Ebenman B., Emmerson M., Montoya J.M., Olesen J.M., Valido A. & Warren P.H. (2005) Body size in ecological networks. *Trends in Ecology and Evolution*, **20**, 402–409.

Zou X.M., Zucca C.P., Waide R.B. & McDowell W.H. (1995) Long-term influence of deforestation on tree species composition and litter dynamics of a tropical rain forest in Puerto Rico. *Forest Ecology and Management*, **78**, 147–157.

(Manuscript accepted 29 April 2010)
