# Supplementary material for: Consumer-driven nutrient recycling of freshwater decapods: Linking ecological theories and application in integrated multitrophic aquaculture
Source: PLoS One. 2023 Oct 26;18(10):e0262972. doi: 10.1371/journal.pone.0262972 (PMC10602317; doi:10.1371/journal.pone.0262972)
Supplement: Attachment — Submitted filename: Response to reviewers.docx [file pone.0262972.s002.docx]

**RESPONSE TO REVIEWERS**

PONE-D-22-00145

Consumer-driven nutrient recycling of freshwater decapods linking ecological theories and application in integrated multi-trophic aquaculture

PLOS ONE

**Response to academic editor**

Query 1

The questions and hypotheses were a bit confusingly laid out. In particular, questions 1 and 2 seem redundant. Both questions appear to ask how N, P, and N:P excretion are influenced by a list of several predictor variables. I think you may have distinguished these as two questions to match the two different statistical approaches (mixed-effects models vs. ANCOVA regressions). But ultimately, they seem like the same question to me: how do a bunch of variables (body size, etc.) affect excretion chemistry. Perhaps you can simplify your question statements, followed by a list of predicted relationships (what you currently refer to as hypotheses).

Reply to query 1

We reorganized the questions according to the editor's suggestions and focused on analyzing the effect body size, body composition and feeds on nutrient excretion using only the incubation time of 30 minutes and within taxa. Therefore, we narrow down the first two questions to a single one and eliminate the across taxa analyzes. We kept the list of predicted relationships, as appropriated.

Query 2

I agree with both authors that, overall, the manuscript was too long. The Discussion alone is nearly 3500 words. Perhaps you can focus the Discussion text on a few key findings. I share reviewer 1’s suspicion that you have included too much. Perhaps consider just presenting the species-specific analyses. There are strong species differences in excretion and body nutrient content and ratios, and the species are different sizes (and size also predicts excretion and body nutrient content and ratios). I am therefore unsure that your “across species” analyses (figure 1, table 2) are valid.

Reply to query 2

We reduced the length of the manuscript (around 2000 words) considering the suggestions of editor and reviewers and tried to focus only in key findings. We decided to eliminate the different scales of analysis (across, among, within taxa), and only performed within and among taxa analyses with the appropriate statistical analysis. We simplified the correlations among variables through collinearity tests and performed the posterior analysis according to these results. It is also important to highlight that we focus the within taxa analyses but, according to the aim of using these decapods in integrated aquaculture systems, it is important to us to carry out the analysis among taxa. These changes simplified the results and thus the overall size of the manuscript.

Query 3

Another concern I have with the “across species” analyses is that you detect an effect of species identity using ANCOVA and don’t consider interactions between species identity and body size. But the graphs clearly show that species identity and body size interact (i.e., different slopes over body size for the different species). If you only present species-specific analyses, your story about body size may disappear or be diminished. That would nevertheless be interesting, as it would suggest that the body-size principle of MTE applies more strongly across species than among individuals within a species.

Reply to query 3

As mentioned before, we eliminated the different scales of analysis and, therefore, the across taxa analyses. We now performed the “among taxa” with appropriate statistical analysis. Firstly, we tested the assumption of ANCOVA (as equality slope and covariate between groups). Because the assumptions were not met (i.e. different body mass of decapod species), we ran ANOVA or Wilcoxon tests.

Query 4

In your mixed-effects models, please address the possibility that you have inflated your sample size for all variables besides ‘time’. Each decapod (and all the predictors that go with it, like its body size and body nutrient composition) will be present as two rows in your data table because you have two time points (30 and 60 minutes). You might just state that you did some preliminary analyses showing higher excretion values at 30 minutes, and then proceed with only analyzing the 30-minute data.

Reply to query 4

We performed a preliminary analysis to evaluate the effect of time-lapse incubation (30 and 60 minutes) on the excretion of nutrients using repeated measures ANOVA. We finally selected the 30 minutes data to perform the subsequent analyses.

Query 5

I have great admiration for people who write outside of their native language. It is quite remarkable. Your English grammar is, for the most part, fine. However, your word tense or vocabulary choice is sometimes a bit off, so I suggest getting some help with that.

Reply to query 5

We send the manuscript to revise the vocabulary before the submission to the journal. However, considering the recommendations of editor and reviewer, we remark this comments to the English reviewer, who realized another revision in the new version of the manuscript.

**Response to reviewers**

**Reviewer #1**

Query 6

I have reviewed the manuscript titled "Consumer-driven nutrient recycling of freshwater decapods: linking ecological theories and application in integrated multi-trophic aquaculture. The questions, goals, and objectives and hypotheses identified for the study and laid out in the manuscript are scientifically well justified, however, as a consumer of the information, overall I found the manuscript, especially the description and presentation of the statistical findings, lengthy and hard to follow. Overall, I wonder if the authors tried to do to much in a single manuscript and could have been better served by breaking the manuscript into at least two. There were time, especially for the results and text associated with Tables 2-3 that either I didn't understand this statistical approach, or there were inconsistencies in text from the tables. Second, I found that the overall manuscript was very long. I am not sure if this was due to what all was being covered in the results, or the authors writing style.

Reply to query 6

It is true that the original version of the manuscript was long and difficult to follow. Following the recommendations of the editor and reviewers, we decided to omit some analyzes previously carried out (across taxa analyses) and rearranged others (within and among taxa). In addition, we reduce parts of the text to fit the new analysis approach and focus on discussing the most important findings. Regarding Tables 2 and 3, they were deleted because we focused in the within taxa analysis and, the model selection were made after correlation test. This new analysis approach simplified the results and reduced the overall length of the manuscript.

Query 7

Third, I found that the manuscript might benefit from some more traditional word uses (e.g. "conserved" versus "preserved" line 226), some awkward sentences, correcting the flipping back and forth between decapod and crustaceans (I would use decapods for broad experimental group, unless you mean to refer to crustaceans in genera), replacement of pronouns and ambiguous terms like this, it, etc., and because of the use of numbered citations, avoid using the inference of authors names and use more active sentences (like line 86 " For example, [25] found....), and the removal of unnecessary words or sentences.

Reply to query 7

We followed all recommendations suggested by reviewer 1, both in relation to the use of the words decapods and crustaceans and with respect to the English wording. In the latter case, despite the fact that the manuscript had been previously reviewed, we resubmitted it to an English editing service, highlighting all the suggestions made by the editor and reviewers.

Query 8

Fourth, would it make sense to report the PCA first in the results as it is an exploratory data analysis tool, or did you do that at the end based on the results of the univariate stats?

Reply to query 8

We decided to use the PCA as a conclusion of the overall results and as a way to facilitate the interpretation by lectors. In addition, the PCA data did not include the types of feed once it was previously discarded in linear models.

Query 9

Fifth, would the start of the discussion benefit by a paragraph of identifying the 3-5 main findings of the study to help focus the reader and simply refer to how the finding supported or did not support your expectations.

Reply to query 9

We omitted the first paragraph of the discussion and replaced it with a new, more concise version of the main findings of the study that we had previously redacted in the subsequent paragraphs.

**Reviewer #2**

Query 10

The introduction part is too long, and there are too many hypothesizes that I cannot catch the main research question in this study. The introduction tells the audiences with the significance of the study, the gap between the published works and your study, a clear objective of the research, and the research questions. You may get some ideas of scientific writing from the following article:
Kevin W. Plaxco, 2010. The art of writing science. Protein Science, 19:2261—226. DOI: 10.1002/pro.514

Reply to query 10

We appreciate the recommendation of the article. As mentioned previously, we reduced the length of the manuscript, including the introduction, considering the suggestions of editor and reviewers and tried to focus only in key findings. The elimination of the across taxa analysis and the rearrangement of others simplified the results and thus the overall size of the manuscript.

Query 11

In the MM part, I was confused if you fed the tested three species with both DF and OF fish-food or fed each species with the selected food? The N:P of the two selected food type was similar (3.45 vs 3.06, calculated from Table 1), so why you decided to use these foods?
Reply to query 11

We modified the MM text to avoid confusion the following sentence: “Two commercial feeds (Garay SRL) were used and offered separately”. We decided to use these feeds because they are commercial and locally available for the culture of fish with omnivorous and detritivorous trophic habits. This has to do with our goal of using these decapods in integrated aquaculture with fish, which in turn are fed with these feeds. The N:P ratio of theses feeds are calculated using molar values. Therefore, the correct values for this ratio is 17.7 for OF and 11.9 for DF. We decided to include in Table 1 the molar values of nutrient ratios and delete the analysis realized between OF and DF composition. This was due to our aim of evaluate the effect of feed on excretion rates, and not evaluate the differences of feed composition. Type of feed was included as categorical and not as continuous variable in analysis. In addition, the data of feed composition was not enough to infer reliable statistical differences.

Query 12

For a given species of aquatic consumers, the excretion rates of both N and P, and the excreted N:P were largely determined by the food types (N, P contents and N:P).
What’s the stoichiometric metrics of the three tested species in the present study? Does the N:P varies significantly among them?

Reply to query 12

We interpret that reviewer 2 asks us about the stoichiometry of the body content of decapods. These data are explained in figure 4, in which it is also observed that all decapod species differ among them in terms of body content.

Query 13

The commonly used method for measuring the excretion rates of aquatic animals is directly measure the rate in the lab immediately after collecting the animals from their natural habitats. Did you measure the natural excretion rate of the tested species?
Reply to query 13

We know that this type of approach is commonly used in excretion rates studies. However, our aim was to evaluate it experimentally since in the environment they do not consume artificial feeds. Perhaps in the future we can consider evaluating the excretion rate of these decapods in the field.

Query 14

Other minor comments:
Check the format of citations, some have doi links.
Incorrect unit in the Y axis of Fig. 2b.
Reorganize the Result section.

Reply to query 14

We checked and corrected all the suggestions realized by reviewer 2.
