# Supplementary material for: Consumer-driven nutrient recycling of freshwater decapods: Linking ecological theories and application in integrated multitrophic aquaculture
Source: PLoS One. 2023 Oct 26;18(10):e0262972. doi: 10.1371/journal.pone.0262972 (PMC10602317; doi:10.1371/journal.pone.0262972)
Supplement: Attachment — Submitted filename: Response to editor and reviewers.docx [file pone.0262972.s005.docx]

**RESPONSE TO REVIEWERS**

PONE-D-22-00145R1

Consumer-driven nutrient recycling of freshwater decapods: linking ecological theories and application in integrated multi-trophic aquaculture

PLOS ONE

**Response to academic editor**

Query 1

In the within-species analysis, please better describe the statistical modeling approach. It seems that some pre-screening was done with the Spearman correlations before the full models were fit, but I don’t really understand the overall modeling strategy. Please clarify the sequence of steps. Additionally, please clarify why some models (e.g., *A. uruguayana* N excretion) included the diet*body mass interaction while others did not. I think this explanation is in your writing, but I couldn’t understand it.

Response to query 1

We described in more detail the statistical modeling approach. Anyway, when we checked the Spearman correlations, we realized that it was not the better or the only way to selected variables to then run definitive models. We are very grateful for this suggestion because it allowed us to improve the analysis methodology. Initially, we run correlations test to verify the correlations between independent variables (body mass and body elemental content) and not for select the better model. Regarding the pair of variables that presented collinearities and significance, only one variable of each pair was selected as an independent variable for posterior analysis. In addition, we want to notify that depending on the assumptions of normality and homoscedasticity of the variables, we run Spearman o Pearson correlations. For select the best models to respond the variation of excretion rates, we considered that Akaike information criterion (AIC) was the most appropriate method. Therefore, after testing for collinearities, we chosen the linear models for each species based on AIC, starting (for all species) with the interaction between type of feed and body mass, the interaction between type of feed and body elemental content, and the interaction between body elemental content and body mass (~Feed*Body mass + Feed*Body content + Body content*Body mass). Then, we run the selected models to test the significance of the variables. Moreover, we worked with dummies variables.

When the general model of excretion rate showed many NA (not available) data, we could not run the model with AIC. Then, we separate the general model in small models with the possible combinations of independent interactions and run separately.

Query 2

Please consider the constraints and artifacts of your study design. Reviewer 4 makes some important comments (e.g., about stress to the animals) that you should address.

Response to Query 2

Please, verify the query 16.

Query 3

Acknowledge the lack of nitrite + nitrate data in your excretion analysis, and the lack of fecal egestion data, and how this may constrain your certainty in your conclusions about organism retention vs. recycling of nutrients.

Response to query 3

Please, verify the query 13

Query 4

Check units, as reviewer 4 suggests in several comments.

Response to query 4

We reviewed and correct all units suggested by reviewer 4. Please check the queries 15, 17 and 19.

Query 5

Check calculations of stoichiometric molar ratios, as reviewer 4 suggests. I also noticed this problem while reading the paper. For example, consider the *M. borellii* data in figure 3. Median N is about 1.4. Median P is about 0.04. So molar N:P should be about 70-80 ([1.4/14]/[0.04/31]). But if you look at panel 3C, molar N:P is displayed as about 1. Also, if *M. borellii* has the highest N excretion and lowest P excretion of the three species, it should have the highest excretion N:P. In summary, please thoroughly review your stoichiometric calculations in your original data, and in all analyses, graphs, and tables in this manuscript.

Response to query 5

We thoroughly reviewed our calculations in the original data and found some mistakes. We noticed that the calculations of all figures with ratio values were previously made in µg, not in molar (Figure 2a, b, Figure 3c, and 4 d, e, f). In addition, we log-10 transformed the values of excretion rates among taxa (Figure 3) due to the data scale. Initially, the statistical calculations were made with the correct values. In addition, we found that N:P in the original data sheet had mistaken values due to “copy and taste” errors. These values were corrected and know represent the adequate ratios of nutrient excretion on N:P in *M. borellii*.

Query 6

Hypothesis 2a (line 132) contradicts lines 62-64. Please reconcile.

Response to query 6

We corrected line 63. Where we said that body P content should be **less** in small-bodied organisms, should say that body P content should be **high** in small-bodied organisms

Query 7

In some cases, terms that were significant in the linear model were not significant when evaluated alone (e.g., in regression; lines 306-308, 329-330). Please consider how to reconcile those differences. Alternatively, you might omit the single-factor regressions and adopt the multi-factor linear models as your definitive results.

Response to query 7

We decided to omit the single-factor regressions and adopt the multi-factor linear models as definitive results. We only run linear models (without posterior analysis, as single-factor regressions and ancova) because they are more robust to evaluate a group of variables together, and select those with more predictive power. This decision changed partially the results of within taxa analysis and, consequently, we had to modify partially the text in the results and discussion sections.

Query 8

One shining result in your paper is the PCA. Despite the mixed messages from the other analyses, the PCA very nicely shows the three species separating in multivariate space. I suggest you draw on those findings more in your discussion. Additionally, please introduce the PCA in the data analysis section of the Methods.

Response to query 8

We introduced the PCA in the data analysis section of the Methods. In accordance with this suggestion, we wrote in the second paragraph of the discussion, and when appropriate, the main findings of PCA with respect the three species separation in multivariate space.

Query 9

Please take particular note of reviewer 4’s final two comments. There is a large literature that you could use to more fully contextualize your findings.

Response to query 9

Please, verify the query 22.

Query 10

Writing outside your native language is very admirable. It is not something I could do. However, I think your manuscript could improve with editing by a native English speaker. This might clear up some of the uncertainties I noted above in your statistical analysis.

Response to query 10

Certainly, writing in the non-native language is a challenge but we tried to do our best in each written sentence. If there are words or terms that raise doubts about their meaning, we greatly appreciate that you or the reviewers highlight them, so it is easier, for the English editor and us, to correct those errors. Every time we send the manuscript to review the English, we have a cost to pay, in addition to the time needed, that differs if it is the entire text or a part of it. We appreciate you considering it. If the manuscript now get to a point where acceptance seems more probable, we expected that the editor could lend a hand with finding the unclear sections. With respect the statistical analysis, the uncertainties you noticed probably were clarified with the new statistical description in the methodology (AIC included and Multifactor Linear Models as definitive results).

Query 11

Lastly, please indicate how data will be made available upon publication (e.g., in the EDI data repository)

Response to query 11

Data will be available at CONICET data repository (https://ri.conicet.gov.ar/).

**Response to reviewers**

Query 12

I did not catch from the manuscript the number of organisms incubated in the different treatments and a general experimental scheme. The experiment itself is not complex, but schemes help.

Response to query 12

We decided not to carry out a scheme because, as the reviewer commented, the experimental design is not complex. Furthermore, we do not want to increase the length of the manuscript with information that can be well interpreted from the text. However, we failed to clarify the number of individuals of each species that consumed each of the two feeds offered. For this reason, we made a small modification in the text in order to improve the understanding of the experimental design. The change can be visualized in 190 and 191 of the document without track change.

Query 13

Nitrate and nitrite are also inorganic nitrogen forms. I know they are not metabolic wastes but this is not a reason to avoid their measurements. Microbes growing inside or outside decapods can include nitrifiers, oxidizing the ammonium excreted. This was likely not considered but should be shortly discussed.

Response to query 13

Before initiated the trial, we washed all chambers with bleach (reducing the bacteria load) before the test and filled it with filtered water from the same source. We now included in Mat and Met this clarification to emphasize the rigorous care with excretion chambers conditions previously to the trials began. In line 198 we wrote: “Then, each organism was removed from the plastic recipients, washed with distilled water, and transferred to glass bottles **(previously cleaned with bleach to reduce the bacteria load)** with 150 ml of filtered (MG-F 0.7 µm, Munktell Filter-Sweden) and dechlorinated tap water.” This standardization reduces the variability that may occur due to the nitrifying activity that could occur in 30 minutes. In addition, the measurement of this oxidized N- compounds is not a common methodology observed in the vast existing literature on the same subject. Mcmanamay et al. (2011) is one of the few authors that measured nitrate in the excretion bags, in case of rapid nitrification, and observed that N and P concentration did not change significantly during the incubation time. In this context, we added a brief discussion about this issue in lines 454-457. “In addition, because we did not measured nitrates, 30 minutes of incubation plus standardization of methodology could reduce the error of estimating N excretion due to the action of nitrifying bacteria that oxidize ammonia to nitrites and nitrates [15].”

Query 14

In Table 1 reported molar C:N:P ratios are (very) different from those I calculated from available data (e.g. molar C:N=gC/12:gN:14). It is possible that I am wrong but I ask authors to clarify how they did the calculations.

Response to query 14

Thank you very much for reviewing the values in the table. With this comment we were able to verify that there was an error in the calculations that was due to the fact that the value of g/mol was being multiplied by the value obtained (g/100g) of each element, and not divided, as it should be. The results of Table 1 are now correct.

Query 15

Line 167 Units of conductivity are microS/cm

Response to query 15

We corrected the unity of conductivity

Query 16

The method described in line 195 seems very stressfull for macrofauna. In a time-serie experiment I do not understand the need for washing animals with distilled water: why not to perform a simple transfer of decapods to microcosms without fish feed containing the same pre-incubation water and start measurements, after a T0?

Response to query 16

Rinsing with distilled water was performed very quickly using a sink while the animal was transferred from one container to another. We decided to carry out this procedure to avoid carrying food remains and other compounds that could be in the pre-incubation chamber. We believe that a “shower” of distilled water has not exacerbated the stress that animals suffer due to the experimental manipulation. In addition, we acclimated the individuals to the experimental conditions, which includes the manipulation during feeding time. In any case, the same procedure was performed with all the specimens, which makes the results obtained with similar error and, therefore, comparable.

Query 17

Lines 283-285: I guess correct units are mg ind-1

Response to query 17

We corrected the units of body mass.

Query 18

Line 279-282: Please check calculations in Table 1

Response to query 18

We checked and corrected the calculations in Table 1. In lines285 and 286, we corrected the text once C:P was not similar between OF and DF.

Query 19

Figs. 1 and 3: the units of excretion rates should be reported as micrograms N or P per mg dry mass per hour and not per 0.5 h. “half hour interval” is never reported as excretion time unit in the literature.

Response to query 19

This suggestion was considered and the excretion rates values were expressed by hour in the figures, but the measures were taken at 30 minutes (this clarification was included in the legends).

Query 20

Line 366 and Figure 4. Again I tried to calculate molar N:P, C:N and C:P ratios and what is reported in the graphs on the right dos not match with calculations done with the numbers reported in the left column. Please report how these calculations were carried out. As an example the approximate N:P ratio of M. borellii is (10:14)/(1.3/31)=17 but the graph reports a numbler close to 7.5.

Response to query 20

Again, thank you very much for reviewing the molar values on the graphs. What happened is that at the time of graphing the results we used the data of % (g/100g) and not of molar. We correct the graphs with the ratio in molar unit.

Query 21

I see in the discussion a very limited (no) comparison of rates and elemental composition with data from the literature, to validate what was measured in this work. Diet is different in nature, but numbers/differences should be comparable. If data are not compared the study is self-referential and local.

Response to query 21

We added to the discussion (Nutrient excretion - among taxa) a paragraph about the difficult to compare nutrient excretion rates among different species, methodologies, and unities of measure (lines 547-563). We also added relevant data about body content of other prawn species (we did not found information about others anomurans and crbas) (lines 593-600).

Query 22

In the whole manuscript feaces as a way to get rid of metabolic waste are not cited, but some of the differences among organisms might be due to release of metabolis waste not via excretion but via feaces. The liteature reports many examples about N and P as dissolved or particulate forms. This needs to be discussed and affects also the entire last paragraph of the discussion. In my opinion this last paragraph exceeds what can be inferred from the reported measurements. There is a vast literature on how organisms as decapods can be integrated in fish aquacolture; here this is explored on the very surface.

Response to query 22

We did take into account the nutrients eliminated through egestion and we included it in the discussion previously (line 534 and 647). Of course the literature reports many examples about N and P as dissolved or particulate forms, but our results are focused on the excretion/mineralisation of inorganic nutrients and it would be to extend the discussion towards topics that exceed the objectives of the present work. However, to attain the reviewer's comment, we decided to expand briefly the discussion regarding this topic (lines 488-492).

The last paragraph of the discussion is a brief conclusion of the main findings and what studies are needed to further understand the role of this decapods as animal-mediated nutrient cycling. Considering this, we do not understand why, in your opinion, the discussion of nutrient egestion (via faeces) affects the entire last paragraph.

However, we believe that at some point the reviewer refers to the last item of the discussion (Comments about application in IMTA), not the last paragraph. In this section we added bibliography of recent studies (among the few available) that refer to the culture of a prawn of the genus *Macrobrachium* (as non-fed species, not the main cultured species) with other fish species in an IMTA context (lines 613-625). The existing information is much broader for the species of crustaceans that are fed (main cultured species), that is, those that do not act as waste recyclers.
