# Supplementary material for: Consumer-driven nutrient recycling of freshwater decapods: Linking ecological theories and application in integrated multitrophic aquaculture
Source: PLoS One. 2023 Oct 26;18(10):e0262972. doi: 10.1371/journal.pone.0262972 (PMC10602317; doi:10.1371/journal.pone.0262972)
Supplement: Attachment — Submitted filename: Response to editor.docx [file pone.0262972.s006.docx]

**RESPONSE TO EDITOR**

PONE-D-22-00145R2

Consumer-driven nutrient recycling of freshwater decapods: linking ecological theories and application in integrated multi-trophic aquaculture

**1. L 22: Change “allometrically within taxa” to “allometrically with body mass” (which I think you meant)**

We changed the text to “allometrically with body mass within taxa”.

**2. L 25: Change sentence to read: “Feed interacted with body mass to explain P excretion…**”

We changed the text to “Feed interacted with body mass to explain P excretion of anomurans and N excretion of crabs”.

**3. L 29: Delete “by”**

We deleted it.

**4. L 96: Change “resembles” to “reflects”**

We changed it.

**5. L 97: Change “they could” to “their diet could”**

We changed it.

**6. L 97: Change “in addition” to “and in response”**

We changed it.

**7. L 101: Change “wide” to “substantial”**

We changed it.

**8. L 111: Change “potentiality” to “potential”**

We changed it.

**9. L 124: Change “of” to “as”**

We changed it.

**10. Are hypotheses 1a and 1b meaningfully different? They both seem to predict that N and P excretion correlate with body mass. Perhaps you could delete one of them.**

It is true that they seem very similar. However, while hypothesis 1a affirms that body mass is the variable that best explain nutrient excretion, 1b should state the slope of this relation (negative allometry according to literature). Therefore, we rewrote this hypothesis as follow: “N and P excretion rates negatively scale with body mass”.

**11. In hypothesis 3a, you propose that body C:N decreases in lower trophic positioned species. This seems to contradict the sentence on lines 82-84, in which you imply that C:N decreases at higher (predator) trophic positions. Please resolve or explain this seeming discrepancy.**

We resolved the discrepancy and changed the text to “Body C:N content increases in carcinized decapods and decreases in higher trophic positioned species”

**12. L 136 (Hyp 3a): This hypothesis is unclear with regard to N:P. Is N:P going up or down in carcinized decapods and/or at lower trophic positions?**

We clarified this question rewriting the sentence to “Body C:N content increases in carcinized decapods and decreases in higher trophic positioned species, while N:P increases in more carnivorous species”

**13. Table 1: Remove “%” from the header. You already provide g/100g as units.**

We removed it.

**14. L 192-3: “arranged randomly and interspecified…” – I do not know what you mean by “interspecified”. I think you can just delete “and interspecified”.**

We deleted it.

**15. L 241: Change “analyses” to “analyze”**

We changed it.

**16. L 243: Change “as dummy variable” to “as an indicator variable”. And then state which feed type was 1 and which was 0. Knowing which was 1 and 0 will help readers interpret effects in the model.**

This was added and corrected. Also, it was indicated in the table 2 as “DF” (feed type 1).

**17. L 244: I understand that you evaluated collinearity among body mass and body elemental content because they were both predictor variables and you didn’t want to include two collinear variables as predictors in the same model. However, I do not understand why you examined collinearity among N, P and N:P excretion. These are response variables, all in separate models, so collinearity among them doesn’t really matter.**

The collinearity among N, P and N:P excretion was deleted to the sentence.

**18. General comment about modeling. You only present the “final” model in Table 2. I assume you had some full model (e.g., feed, body mass, body element content, all possible 2-term interactions), and then you fit all possible subsets of that full model to find the one with the lowest AIC. Is this correct? If so, please explain that.**

This was better explained in the manuscript. Also, we forget clarify that we used stepAIC function of the MASS package to selected the best fit models. But this was included now.

**Also, in supplemental information, please provide the results of all model subset fits with some diagnostic criteria (e.g., AIC, R2), perhaps as a table.**

We provided the results of AIC of all model subset using stepAIC function in a table as supplemental information.

**19. L 274-5: You state that you log-transformed variables to meet assumptions of normality. This seems to slightly contradict the previous paragraph (L 269-272), which implies that sometimes the assumptions of normality were not met, and so non-parametric statistics were used (in which case, I assume you would not log transform, as there would be no reason to in rank-based non-parametric analyses). Do you really mean that you log-transformed, and if the assumptions of normality were still not met after transforming, then you used non-parametric stats?**

This was clarified in each paragraph of this section of the manuscript: In within and among taxa analysis, the variables used were log10-transformed to meet assumptions of normality and heterogeneity of variances. In addition, for working with linear relationships between quantitative variables, normality and homoscedasticity assumptions were tested on the models. In within taxa analysis, if the assumptions of normality and homoscedasticity were still not met after transforming, data without log10-transformed were used in non-parametric stats. Wilcoxon pairwise test was run if the assumptions of normality and homoscedasticity were not met in ANCOVA model. If these assumptions were met, but ANCOVA assumptions was not met (different slopes and covariate), one-way ANOVA with Tukey pairwise was run.

**20. L 297: Change “more N at” to “N faster over”**

We changed it.

**21. L 298: Change “at 60” to “over 60”**

We changed it.

**22. L 309: At end of line, change “any” to “no”**

We changed it.

**23. L 311-312: On L 311 you say there was collinearity (implying a significant relationship), but on L 312, you note p > 0.05**

We corrected it.

**24. L 329-330: These results are an example of when it would be helpful to know which feed type =1 and which type =0 in your indicator (“dummy”) variable**

We clarified it in data analysis.

**25. L 332: Why were some data NA for N:P? Was it because P = 0 (i.e., below detection limit), so the ratio can’t be calculated?**

What happens is that there is little data of the N:P body content of *T. borellianus*. We had a technical problem when we sent the samples to determine elemental composition. The required gas used in the atmosphere of the elemental analyser ran out, which implied that the analyzer returned unrealistic values for N in several samples and we lost samples. The amount of samples that we had was small (200 mg of sample is required for a duplicate determination) and therefore we could not repeat the analyses. So, some individuals remained with P values but not with N values and, for this reason, we calculated N:P values only in few individuals. Then, when we run the full model with several interactions, the number of NA data reduced the freedom degrees with respect the number of variables and the model failed. This was clarified in the manuscript.

**26. L 334: “run separately” – Do you mean that you fit several single-factor models of N:P vs. one predictor variable? If so, please clarify.**

We mean that several models of N:P vs. each interaction predictor variable were run (N:P vs. Feed*Body mas; N:P vs. N:P body*Body mass; N:P vs. Feed*N:P body). We clarified this in the manuscript. In this way, the independent variables that interact are reduced and therefore the degrees of freedom.

**27. L 335: Change “differences were showed” to “effects were identified”**

We changed it.

**28. Table 2: Include actual coefficient values, not just the sign. This way, readers can identify the magnitude by which an interaction term affects the slope of a factor.**

This was included in the table 2. Some of the statistical values changed but the relationships did not. This is because we had to double the values for excretion rate in 30 minutes (following the suggestion of one reviewer) so that it can be expressed per hour and not half an hour.

**29. Table 2 – A. uruguayana – Excretion N: Change “DF*Body mass” to “Feed*Body mass”.**

We changed it.

**30. L 347-349: It appears that you’re reporting whether body element content and stoichiometric ratios were correlated with body mass. But I do not understand why you say there were no “significant linear relations and neither collinearities”. Why make reference to both linear relations and collinearities? Are you reporting two different groups of analyses here?**

We reported two different groups of analyses and this was included and clarified in data analysis section.

**31. Please start your Discussion with a straightforward “take-home” message about whether each hypothesis was supported, and if so, by which species or nutrient. This will help your readers link your findings back to your hypotheses. You could even organize this as a table if that helps you.**

We started the discussion with a take-home message followed by answering each hypothesis and prediction made in the introduction. This certainly will help readers to link results with hypothesis.

**32. L 462: Change “MET” to “MTE”**

We changed it.

**33. L 472: Change “found” to “find”**

We changed it.

**34. L 483: Delete “were”**

We deleted it.

**35. L 484: I do not understand the references to negative and positive allometry when discussing effects of feed type.**

Negative and positive allometry is referred to the slope of the interaction between body mass and nutrient excretion. To avoid confusion, we decided to change the rewrite the test as follows: Regardless of the biomass, anomurans excreted more P and crabs excreted more N when fed OF. When body mass interacted with excretion, anomurans had negative allometry for P excretion and crabs positive allometry for N excretion.

**36. L 493: Add “to” after “respect” (“With respect to…”)**

We added it.

**37. L 494: Change “to” to “for”**

We changed it.

**38. L 497: Add “to” after “feasible” (“feasible to collect…”)**

We added it

**39. L 522-523: Rewrite as “…main factors separating the species in multivariate space.”**

We rewrote it.

**40. L 523: Change “was” to “were”**

We changed it.

**41. L 549: Change “unities” to “units”**

We changed it.

**42. L 556: Make the “4” a subscript in “NH4-N”**

We subscribed it.

**43. L 560: Change “Despite” to “Although”**

We changed it.

**44. L 560: Change “unity” to “units”**

We changed it.

**45. L 562: Change “unities” to “units”**

We changed it.

**46. L 566: Delete “due to the”**

We deleted it.

**47. L 566: Change “content” to “contents”**

We changed it.

**48. Be sure to upload high quality figures. They are a little fuzzy in my PDF copy**

The figures were in good quality when we uploaded it. However, we modified them again according to the dimension specifications (2250 pixels of width maximum and 2625 pixels of height maximum, both at 300 dpi). Before these modifications, figures had lower pixels of width and height but higher dpi (600). Likewise, I think that the pdf converter lowers the quality of the image and that is why it looks fuzzy. The original looks great.

**Journal Requirements:**

We added a new cite for an statistic procedure. It is with the number 85 - Ripley B, Venables B, Bates DM, Hornik K, Gebhardt A, Firth D, Ripley MB. Package ‘mass’. Cran r, 2013: 538, 113-120 .
